# Lifestyles, metabolome and diabetic kidney disease: a cohort study

**DOI:** 10.1093/qjmed/hcaf281

**Published:** 2025-11-17

**Authors:** Bowen Deng, Yidan Zheng, Zihao Zhou, Li Xu, Fei Li, Chun Zhang

**Affiliations:** Department of Nephrology, Union Hospital, Tongji Medical College, Huazhong University of Science and Technology, Wuhan, China; Department of Cardiovascular Surgery, Union Hospital, Tongji Medical College, Huazhong University of Science and Technology, Wuhan, China; Department of Cardiovascular Surgery, Union Hospital, Tongji Medical College, Huazhong University of Science and Technology, Wuhan, China; Department of Cardiovascular Surgery, Union Hospital, Tongji Medical College, Huazhong University of Science and Technology, Wuhan, China; Department of Cardiovascular Surgery, Union Hospital, Tongji Medical College, Huazhong University of Science and Technology, Wuhan, China; Department of Structural Heart Disease, Yunnan Fuwai Cardiovascular Hospital, Kunming Medical University, Kunming, China; Department of Nephrology, Union Hospital, Tongji Medical College, Huazhong University of Science and Technology, Wuhan, China

## Abstract

**Background:**

Diabetic kidney disease (DKD) is a leading cause of kidney failure closely linked to lifestyle factors, but the mechanisms have not been systematically investigated.

**Aim:**

This study aimed to assess the long-term metabolic effects of lifestyle behaviors on DKD.

**Design and methods:**

This study aimed to examine links between lifestyle, metabolic biomarkers, and DKD incidence and mortality in a population with diabetes. This study analyzed data from 18 287 participants, evaluating five lifestyle factors (diet, sleep duration, physical activity, smoking and alcohol intake) alongside 251 metabolic biomarkers. Cox proportional hazards models and Mendelian randomization (MR) assessed associations. Mediation analysis was conducted on biomarkers linked to both lifestyle and DKD. Additionally, genome-wide association study (GWAS) and gene enrichment analysis were conducted on mediating biomarkers to explore biological mechanisms.

**Results:**

Among 18 287 participants with diabetes, 3247 developed DKD over a median follow-up of 14.6 years. Lipids and amino acids were associated with DKD and mediated the effects of lifestyle factors. Mediating biomarkers, including triglycerides to total lipids in HDL percentage and glycoprotein acetyls, demonstrated both observational and causal associations with DKD. The mediation effects differed between various levels of blood glucose control. Pathway enrichment analysis identified both shared and distinct biological pathways.

**Conclusions:**

This comprehensive study underscores the importance of metabolomics in delineating the mechanisms by which lifestyle behaviors influence DKD, paving the way for targeted interventions.

## Introduction

Diabetic kidney disease (DKD) is a major complication of diabetes, leading to end-stage renal disease (ESRD), and its prevalence is increasing globally in parallel with that of diabetes.^1^^,^^2^ Lifestyle factors, including diet, sleep, physical activity, smoking and alcohol intake, influence DKD onset and progression.^3–7^ Metabolomics helps elucidate how these factors affect metabolic pathways and disease progression. Prior studies link lifestyle behaviors to changes in fatty acid and amino acid metabolism, impacting kidney function.^8–14^ However, comprehensive studies integrating these metabolic effects with DKD risk remain limited.

This study employed metabolomics to investigate how diet, sleep, physical activity, smoking and alcohol intake influence metabolic pathways and DKD risk, providing a basis for targeted interventions in prevention and management.

## Methods

### Study population

**Figure 1.**
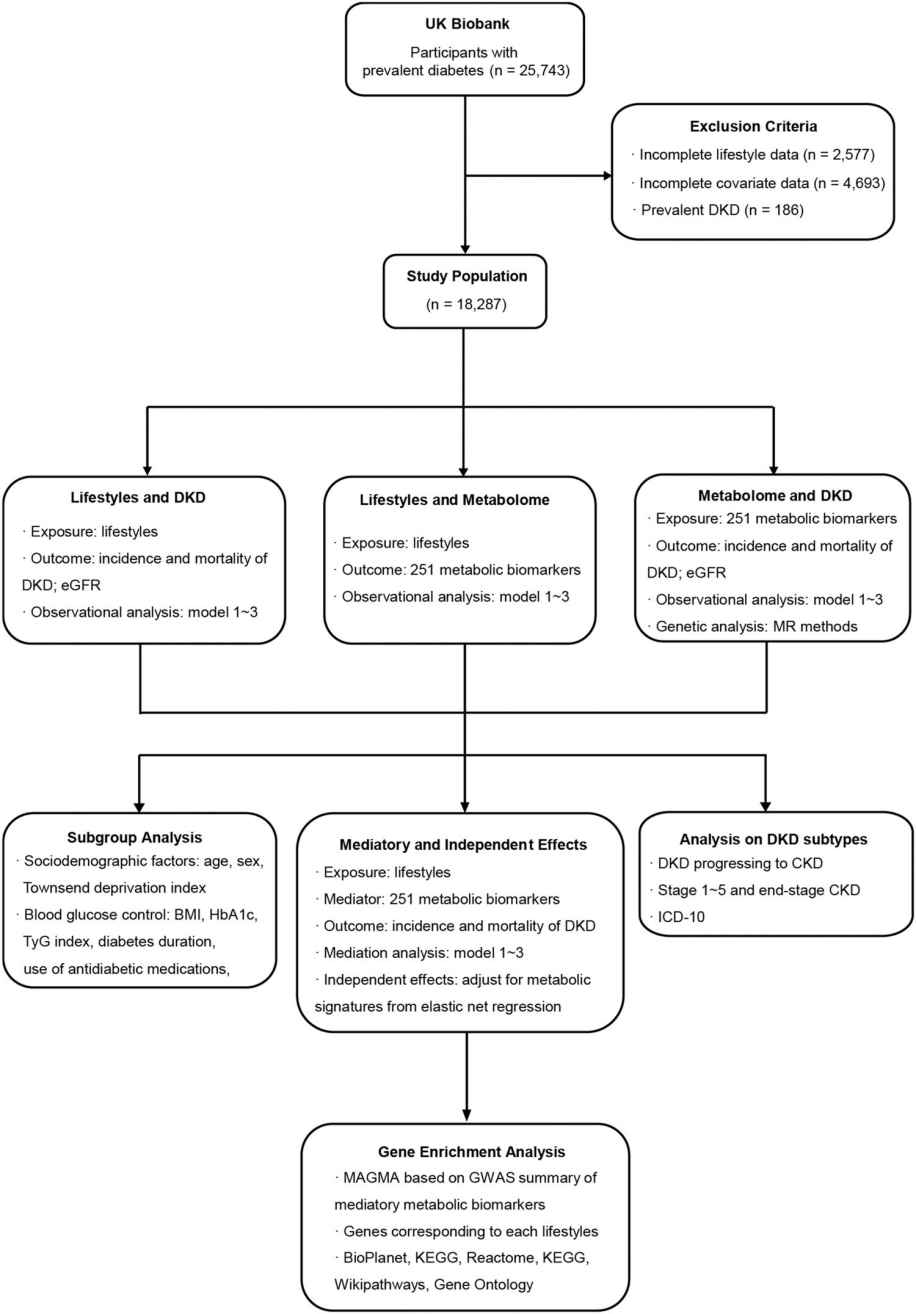
Study design flowchart outlining the analytical approach to investigate the role of metabolomic profiles in the association between lifestyles and diabetic kidney disease (DKD) among UK Biobank participants with diabetes. The study cohort was derived from 25 743 eligible individuals, with exclusions applied for incomplete data (lifestyle or covariates) and prevalent DKD, resulting in a final population of 18 287. The analytical framework comprised three core components: (1) assessing the observational association between lifestyles (exposure) and DKD outcomes (incidence, mortality, and eGFR); (2) evaluating the relationship between lifestyles and 251 circulating metabolic biomarkers; and (3) examining the link between these biomarkers and DKD outcomes, using multivariable-adjusted models (models 1–3) throughout. Subsequent steps included subgroup analyses (by sociodemographic and clinical factors), mediation analysis to test if metabolites mediate the lifestyle–DKD link, analysis of independent lifestyle effects adjusting for metabolic signatures, and evaluation of DKD subtypes. Finally, gene enrichment analysis (MAGMA) was conducted on GWAS summaries of mediating metabolites to identify enriched biological pathways.

The study design is shown in Figure 1. The UK Biobank (UKB) is a large-scale, longitudinal cohort study that enrolled participants aged 40–70 years from the general population between 2006 and 2010 across 22 centers in the UK. Ethical approval was obtained from the North West Multicenter Research Ethics Committee, and all participants provided written informed consent.^15^ The study was conducted under UKB application number 105945. Inclusion criteria for participants were as follows: the presence of diabetes at baseline (defined using International Classification of Diseases, Tenth Revision codes E11, E12, E13, and E14, as well as self-reported medical conditions), absence of DKD at baseline, and availability of complete lifestyle and covariate data (Supplementary Table S1).

### Phenotype definitions

Five lifestyle factors, including diet, sleep duration, physical activity, smoking status and alcohol intake, were assessed in this study. (Detailed definitions are provided in the Supplementary Methods.) Participants with complete metabolomic data of 251 metabolic biomarkers (*n* = 9412) were included for analysis involving metabolic biomarkers. (Analytical details are provided in the Supplementary Methods.) Inverse normal rank transformation was applied to each metabolite to account for batch effects and to normalize the distributions for subsequent analyses. DKD was identified using the International Classification of Diseases, Tenth Revision (ICD-10) codes E11.2, E12.2, E13.2, E14.2, N18.0, N18.1, N18.2, N18.3, N18.4, N18.5, N18.8, and N18.9.^7^ (Details of disease subtype and mortality are provided in the Supplementary Methods and Supplementary Table S1.) Participants were followed from the date of recruitment until the occurrence of DKD diagnosis, death, or the last follow-up date (30 September 2021), whichever came first. Covariates involved in regression models were defined in Supplementary Methods and Supplementary Table S1.

### Statistical analysis

Continuous variables were compared using *t*-tests, and categorical variables with chi-squared or Fisher’s exact tests. Associations between lifestyle factors, metabolic biomarkers, eGFR and DKD were assessed using Cox proportional hazards models and general linear models (GLM), with additional adjustment for metabolic signatures to evaluate independent effects. Mendelian randomization (MR) was applied to examine causal effects, accounting for heterogeneity, pleiotropy and reverse causality, with significance defined as *P* < 0.05. Mediation analysis focused on biomarkers associated with both lifestyles and DKD, and sensitivity analyses included participants with eGFR ≥ 60. eGFR was calculated using the 2021 race-free CKD-EPI equation.^16^ We performed principal component analysis (PCA) and orthogonal projections to latent structures-discriminant analysis (OPLS-DA) for dimensionality reduction and clustering of metabolites,^17–19^ using DKD status and subgroup classifications as grouping variables to visualize clustering results. GWAS summary statistics for mediating biomarkers were obtained from IEU OpenGWAS,^20^ and analyzed with Multi-marker Analysis of Genomic Annotation (MAGMA). Gene enrichment analyses were conducted using BioPlanet, KEGG, MSigDB, Reactome, Wikipathways, and Gene Ontology, with tissue-specific annotation from GTEx v8. False discovery rate correction was applied for multiple comparisons in association and mediation analysis. All analyses were performed in R 4.3.1. Details of the statistical analysis are provided in the Supplementary Methods.

## Results

### Baseline characteristics of participants

After excluding participants with incomplete data or prevalent DKD, 18 287 diabetes patients from the UK Biobank were analyzed (mean age 59.9 years, 36.9% female; median follow-up 14.6 years). During follow-up, 3732 (17.8%) developed DKD and 451 (2.2%) died from DKD. Participants with DKD were older, had higher Townsend deprivation index (TDI), more unhealthy lifestyles, higher BMI, glycated hemoglobin (HbA1c), triglyceride-glucose (TyG) index, longer diabetes duration, more hypertension and more medication use (Table 1; CKD stages in Supplementary Table S4).

**Table 1:** Baseline characteristics of study population

	Control (*N* = 15 040)	DKD cases (*N* = 3247)	*P* value
Age, mean (SD), years	59.4 (7.11)	62.5 (5.68)	<0.001
Sex			0.501
Male	9542 (63.4%)	2081 (64.1%)	
Female	5498 (36.6%)	1166 (35.9%)	
Townsend deprivation index, mean (SD)	−0.66 (3.34)	−0.39 (3.42)	<0.001
White British			0.011
Yes	13 437 (89.3%)	2950 (90.9%)	
No	1603 (10.7%)	297 (9.15%)	
College or university degree:			<0.001
Yes	3914 (26.0%)	599 (18.4%)	
No	11 126 (74.0%)	2648 (81.6%)	
Diet score, mean (SD)	2.69 (1.17)	2.63 (1.15)	0.014
Sleep duration 7–8 h per day			<0.001
Yes	9171 (61.0%)	1821 (56.1%)	
No	5869 (39.0%)	1426 (43.9%)	
Physical activity			<0.001
High activity	658 (4.38%)	87 (2.68%)	
Medium activity	11 763 (78.2%)	2351 (72.4%)	
Low activity	846 (5.62%)	232 (7.15%)	
Never activity	1773 (11.8%)	577 (17.8%)	
No current smoker			0.402
Yes	13 437 (89.3%)	2884 (88.8%)	
No	1603 (10.7%)	363 (11.2%)	
Moderate alcohol intake			0.007
Yes	3614 (24.0%)	707 (21.8%)	
No	11 426 (76.0%)	2540 (78.2%)	
BMI, mean (SD)	31.0 (5.80)	32.4 (5.77)	<0.001
HbA1c, mean (SD)	52.1 (13.4)	55.4 (14.9)	<0.001
Diabetes duration, mean (SD), years	6.22 (8.62)	6.85 (8.69)	<0.001
Prevalence of hypertension			<0.001
Yes	3725 (24.8%)	1396 (43.0%)	
No	11 315 (75.2%)	1851 (57.0%)	
Use of lipid-lowering medication			<0.001
Yes	11 284 (75.0%)	2676 (82.4%)	
No	3756 (25.0%)	571 (17.6%)	
Use of anti-hypertensive medication			<0.001
Yes	9179 (61.0%)	2589 (79.7%)	
No	5861 (39.0%)	658 (20.3%)	
Use of antidiabetic medication:			<0.001
Yes	10 352 (68.8%)	2592 (79.8%)	
No	4688 (31.2%)	655 (20.2%)	
TyG, mean (SD)	5.77 (0.70)	5.93 (0.71)	<0.001

Abbreviations: SD, standard error; BMI, body mass index; HbA1c, glycated hemoglobin; TyG index, triglyceride-glucose index.

### Associations of lifestyles and metabolic biomarkers with risk of incident DKD

**Figure 2.**
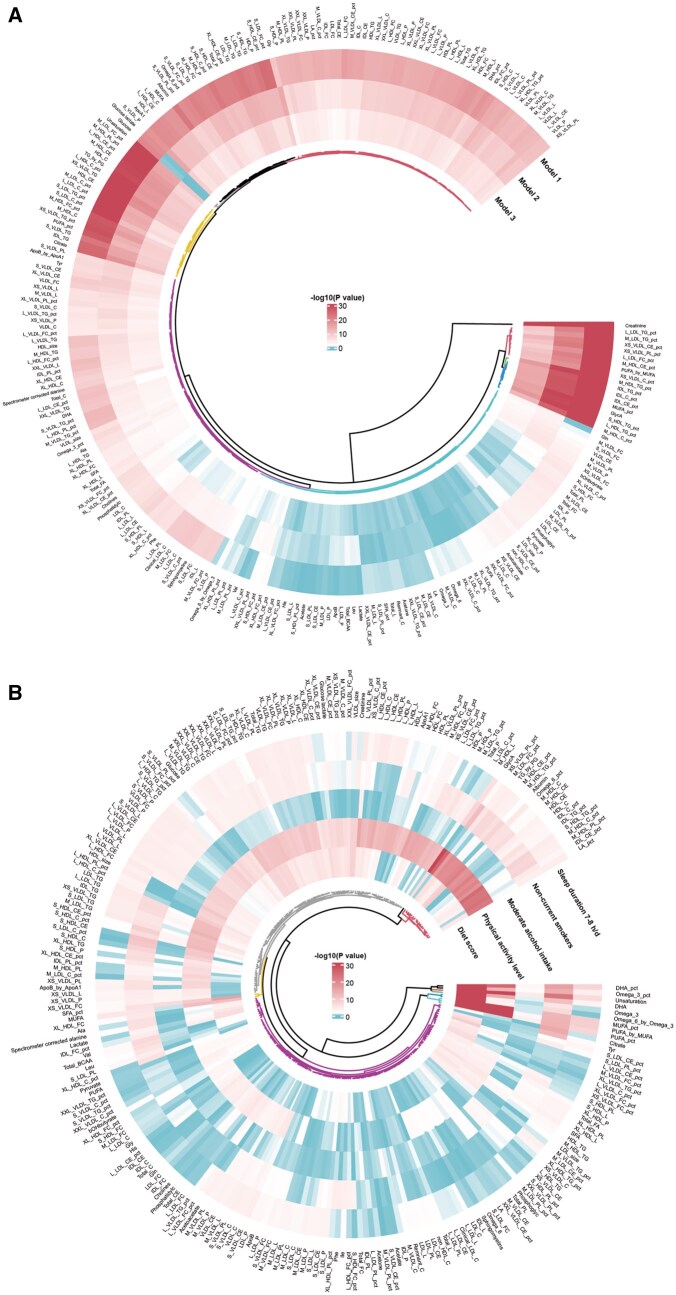
Associations between lifestyles, metabolic biomarkers and DKD incidence in observational analysis. (**A**) The statistical significance of associations between 251 metabolic biomarkers and the risk of DKD across models 1, 2 and 3. Shading intensity represents FDR-adjusted *P* values, with darker shading indicating statistically significant positive or negative associations, and lighter shading indicating non-significant associations. The concentric ring shows the number of clusters derived from hierarchical clustering based on association patterns. Notably, this analysis highlights extensive significant associations of lipid metabolites, particularly triglyceride and total lipid percentages within HDL subclasses, as well as amino acid metabolites with DKD risk. (**B**) The associations between five lifestyle factors and 251 circulating metabolic biomarkers. Shading intensity represents FDR-adjusted *P* values, with darker shading indicating statistically significant positive or negative associations and lighter shading indicating non-significant associations. Dendrograms above and to the left show the hierarchical clustering of lifestyle factors and metabolic biomarkers, respectively. The analysis indicates that diet, sleep, physical activity, and smoking are primarily associated with lipid and amino acid metabolic pathways, whereas alcohol consumption is mainly associated with lipid-related biomarkers.

In model 1, the five lifestyle factors were associated with increased risk of incident DKD, and these associations were consistent in models 2 and 3 (Supplementary Figure S1). Similar trends were observed for eGFR in sensitivity analyses (Supplementary Figure S2). Diet, sleep, physical activity and smoking were also linked to DKD mortality (Supplementary Figure S3). After FDR correction, 208, 194 and 173 metabolic biomarkers were significantly associated with DKD incidence in models 1–3, while 176, 122 and 14 were linked to DKD mortality, mainly involving lipid and amino acid metabolism (Figure 2A; Supplementary Tables S4–5 and Figure S4). Creatinine showed the strongest positive association, validating the genetic instruments. Strong positive associations with incident DKD were observed for triglycerides to total lipids in medium HDL (HR = 1.14; 95% CI, 1.10–1.17; *P* after FDR < 0.001) or large HDL percentage (HR = 1.11; 95% CI, 1.09–1.14; *P* after FDR < 0.001) and glycoprotein acetyls (HR = 1.23; 95% CI, 1.17–1.28; *P* after FDR < 0.001), whereas cholesterol (HR = 0.86; 95% CI, 0.83–0.89; *P* after FDR < 0.001) or cholesteryl esters to total lipids in IDL percentage (HR = 0.85; 95% CI, 0.82–0.88; *P* after FDR < 0.001) showed strong negative associations (model 3; Supplementary Table S4). Most biomarker associations were replicated for eGFR (Supplementary Figure S5 and Table S6).

### Variation of the metabolic biomarkers in response to lifestyles

A total of 154, 159, 200, 161 and 46 metabolic biomarkers were associated with diet, sleep, physical activity, smoking and alcohol intake in model 1 (Figure 2B; Supplementary Table S7). Replicated associations in model 2 were 111, 37, 153, 121 and 34 (Supplementary Figure S6 and Table S7), and in model 3 were 100, 21, 155, 116 and 34 (Supplementary Figure S7 and Table S7). The strongest biomarkers in model 3 were docosahexaenoic acid to total fatty acids percentage for diet (β = 0.14; 95% CI 0.13–0.16; *P* after FDR < 0.001) and sleep (β = 0.06; 95% CI 0.02–0.10; *P* after FDR = 5.23E−3), glycoprotein acetyls for physical activity (β = −0.10; 95% CI −0.13 to −0.08; *P* after FDR < 0.001), degree of unsaturation for smoking (β = −0.36; 95% CI −0.42 to −0.30; *P* after FDR < 0.001) and saturated fatty acids to total fatty acids percentage for alcohol intake (β = 0.11; 95% CI 0.06–0.16; *P* after FDR < 0.001). These associations were consistently replicated across models.

Metabolic biomarkers linked to diet, sleep, physical activity, and smoking were mainly related to lipid metabolism, including fatty acids, cholesterol and lipoproteins, as well as protein and amino acid metabolism, whereas alcohol intake was primarily associated with lipid metabolism. Interaction analysis indicated that the Townsend deprivation index, antidiabetic medication use, and BMI had more significant interactions with metabolic biomarkers (Supplementary Figure S8). PCA-based dimensionality reduction and clustering showed limited group separation, while OPLS-DA revealed more distinct group-specific distributions (Supplementary Figures S9 and 10), likely because OPLS-DA incorporates class information, removing irrelevant variance and highlighting group differences, whereas PCA captures overall variance, which may mask subtle signals.^17–19^

### Mediation analysis

A total of 30, 11, 20, 35 and 6 metabolic biomarkers mediated the associations between diet, sleep, physical activity, smoking and alcohol intake with DKD incidence, with mediation proportions >10% for each factor (Table 2; Supplementary Table S8). For DKD mortality, 11, 20 and 33 biomarkers significantly mediated the effects of sleep, physical activity and smoking (Supplementary Table S9). Most mediated effects involved lipid metabolism, including polyunsaturated to monounsaturated fatty acids ratio and degree of unsaturation, which mediated all lifestyle–DKD associations. Some biomarkers not directly linked to both lifestyle and DKD, such as triglycerides in small LDL, apolipoprotein A1 and free cholesterol in chylomicrons/extremely large VLDL, also mediated effects, e.g. moderate alcohol intake reducing DKD risk.

**Table 2:** Mediation effects of metabolic biomarkers between lifestyles and DKD incidence based on model 1 in observational analysis^a^

Mediators	Proportion	CI lower	CI upper	*P* value
Diet				
Triglycerides in small HDL	0.11	0.05	0.20	<2E−16
Cholesterol to total lipids in large HDL percentage	0.10	0.05	0.24	<2E−16
Cholesteryl esters in chylomicrons and extremely large VLDL	0.11	0.05	0.21	<2E−16
Phospholipids to total lipids in medium HDL percentage	0.10	0.04	0.20	<2E−16
Omega 6 fatty acids to total fatty acids percentage	0.13	0.08	0.27	<2E−16
Triglycerides to phosphoglycerides ratio	0.14	0.08	0.34	<2E−16
Glycoprotein acetyls	0.19	0.10	0.42	<2E−16
Glucose	0.11	0.05	0.25	<2E−16
Glucose lactate	0.11	0.06	0.26	<2E−16
Free cholesterol to total lipids in IDL percentage	0.11	0.06	0.22	<2E−16
Monounsaturated fatty acids to total fatty acids percentage	0.43	0.27	0.86	<2E−16
Docosahexaenoic acid	0.27	0.16	0.51	<2E−16
Omega 3 fatty acids to total fatty acids percentage	0.25	0.13	0.54	<2E−16
Cholesteryl esters to total lipids in medium HDL percentage	0.11	0.05	0.21	<2E−16
Albumin	0.14	0.08	0.28	<2E−16
Phospholipids to total lipids in large VLDL percentage	0.12	0.07	0.22	<2E−16
Cholesteryl esters to total lipids in large HDL percentage	0.12	0.06	0.25	<2E−16
Polyunsaturated fatty acids to total fatty acids percentage	0.32	0.21	0.63	<2E−16
Triglycerides to total lipids in IDL percentage	0.11	0.05	0.22	<2E−16
Cholesteryl esters to total lipids in very small VLDL percentage	0.12	0.06	0.24	<2E−16
Triglycerides to total lipids in small HDL percentage	0.13	0.06	0.24	<2E−16
Cholesterol to total lipids in very small VLDL percentage	0.12	0.07	0.25	<2E−16
Polyunsaturated fatty acids to monounsaturated fatty acids ratio	0.42	0.26	0.91	<2E−16
Docosahexaenoic acid to total fatty acids percentage	0.40	0.22	0.88	<2E−16
Degree of unsaturation	0.50	0.31	1.04	<2E−16
Cholesterol to total lipids in medium HDL percentage	0.13	0.04	0.26	<2E−16
Monounsaturated fatty acids	0.14	0.07	0.26	<2E−16
Cholesteryl esters in large HDL	0.10	0.05	0.27	<2E−16
Free cholesterol to total lipids in medium LDL percentage	0.12	0.07	0.34	<2E−16
Triglycerides to total lipids in very small VLDL percentage	0.11	0.06	0.28	<2E−16
Sleep				
Glycoprotein acetyls	0.15	0.10	0.25	<2E−16
Docosahexaenoic acid to total fatty acids percentage	0.10	0.06	0.20	<2E−16
Polyunsaturated fatty acids to total fatty acids percentage	0.13	0.07	0.22	<2E−16
Monounsaturated fatty acids to total fatty acids percentage	0.19	0.12	0.33	<2E−16
Triglycerides to phosphoglycerides ratio	0.10	0.05	0.17	<2E−16
Polyunsaturated fatty acids to monounsaturated fatty acids ratio	0.17	0.10	0.33	<2E−16
Cholesteryl esters in HDL	0.11	0.05	0.22	<2E−16
Cholesteryl esters in large HDL	0.10	0.05	0.17	<2E−16
Phospholipids to total lipids in large VLDL percentage	0.10	0.05	0.22	<2E−16
Degree of unsaturation	0.14	0.08	0.26	<2E−16
Triglycerides to total lipids in small HDL percentage	0.11	0.07	0.19	<2E−16
Physical activity				
Degree of unsaturation	0.16	0.12	0.21	<2E−16
Phospholipids to total lipids in medium HDL percentage	0.13	0.09	0.18	<2E−16
Cholesteryl esters to total lipids in IDL percentage	0.16	0.12	0.21	<2E−16
Glycoprotein acetyls	0.20	0.16	0.26	<2E−16
Triglycerides to total lipids in small HDL percentage	0.14	0.11	0.20	<2E−16
Monounsaturated fatty acids to total fatty acids percentage	0.20	0.16	0.28	<2E−16
Polyunsaturated fatty acids to total fatty acids percentage	0.14	0.10	0.19	<2E−16
Cholesteryl esters in HDL	0.14	0.10	0.18	<2E−16
Creatinine	0.14	0.10	0.23	<2E−16
Cholesterol in medium HDL	0.12	0.09	0.15	<2E−16
Polyunsaturated fatty acids to monounsaturated fatty acids ratio	0.18	0.14	0.25	<2E−16
Cholesteryl esters to total lipids in very small VLDL percentage	0.11	0.07	0.15	<2E−16
Cholesteryl esters in medium HDL	0.12	0.09	0.17	<2E−16
Phospholipids to total lipids in very small VLDL percentage	0.16	0.12	0.22	<2E−16
Cholesterol to total lipids in IDL percentage	0.15	0.10	0.20	<2E−16
Cholesterol to total lipids in medium HDL percentage	0.13	0.09	0.17	<2E−16
Triglycerides to total lipids in IDL percentage	0.13	0.09	0.18	<2E−16
Cholesteryl esters to total lipids in medium HDL percentage	0.11	0.08	0.16	<2E−16
HDL cholesterol	0.13	0.09	0.17	<2E−16
Triglycerides to phosphoglycerides ratio	0.12	0.08	0.16	<2E−16
Smoking				
Cholesterol to total lipids in medium HDL percentage	0.15	0.09	0.35	<2E−16
Cholesterol to total lipids in IDL percentage	0.16	0.09	0.31	<2E−16
Phospholipids to total lipids in very small VLDL percentage	0.24	0.13	0.49	<2E−16
Polyunsaturated fatty acids to monounsaturated fatty acids ratio	0.24	0.16	0.37	<2E−16
Docosahexaenoic acid	0.11	0.06	0.19	<2E−16
Cholesteryl esters in medium HDL	0.16	0.09	0.29	<2E−16
Docosahexaenoic acid to total fatty acids percentage	0.17	0.10	0.31	<2E−16
Cholesterol in medium HDL	0.15	0.08	0.27	<2E−16
Cholesterol to total lipids in small HDL percentage	0.11	0.07	0.31	<2E−16
Albumin	0.15	0.08	0.27	<2E−16
Cholesteryl esters in small HDL	0.15	0.10	0.31	<2E−16
Cholesterol in small HDL	0.12	0.07	0.19	<2E−16
Polyunsaturated fatty acids to total fatty acids percentage	0.19	0.11	0.34	<2E−16
Omega 6 fatty acids to total fatty acids percentage	0.11	0.06	0.24	<2E−16
Cholesteryl esters to total lipids in IDL percentage	0.15	0.07	0.28	<2E−16
Triglycerides in large LDL	0.11	0.06	0.20	<2E−16
Triglycerides to total lipids in IDL percentage	0.15	0.09	0.30	<2E−16
Concentration of HDL particles	0.12	0.07	0.25	<2E−16
HDL cholesterol	0.11	0.05	0.23	<2E−16
Omega 3 fatty acids to total fatty acids percentage	0.10	0.05	0.17	<2E−16
Monounsaturated fatty acids to total fatty acids percentage	0.27	0.16	0.42	<2E−16
Triglycerides to total lipids in small HDL percentage	0.19	0.10	0.31	<2E−16
Free cholesterol to total lipids in medium LDL percentage	0.11	0.06	0.23	<2E−16
Degree of unsaturation	0.26	0.17	0.59	<2E−16
Cholesteryl esters in HDL	0.14	0.07	0.23	<2E−16
Cholesteryl esters to total lipids in medium HDL percentage	0.16	0.10	0.36	<2E−16
Triglycerides to phosphoglycerides ratio	0.16	0.08	0.27	<2E−16
Glycoprotein acetyls	0.19	0.12	0.40	<2E−16
Triglycerides in IDL	0.11	0.07	0.24	<2E−16
Phospholipids to total lipids in medium HDL percentage	0.16	0.09	0.37	<2E−16
Triglycerides in LDL	0.11	0.06	0.20	<2E−16
Concentration of medium HDL particles	0.10	0.06	0.28	<2E−16
Cholesteryl esters to total lipids in very small VLDL percentage	0.11	0.04	0.21	<2E−16
Cholesteryl esters to total lipids in small HDL percentage	0.14	0.08	0.32	<2E−16
Cholesterol to total lipids in small LDL percentage	0.13	0.08	0.30	<2E−16
Alcohol intake				
Phospholipids to total lipids in large VLDL percentage	0.19	0.07	0.70	<2E−16
Degree of unsaturation	0.26	0.09	1.03	0.04
Polyunsaturated fatty acids to monounsaturated fatty acids ratio	0.17	0.07	1.87	<2E−16
Triglycerides in LDL	0.10	0.03	1.47	0.02
Triglycerides in LDL	0.13	0.02	0.69	0.04
Cholesteryl esters to total lipids in small HDL percentage	0.15	0.04	0.50	0.04

a Metabolic biomarkers that fulfilled the following criteria are presented: (i) significant association with lifestyles; (ii) significant association with DKD incidence; (iii) significant mediation effect; (iv) mediation proportion >10%.

**Figure 3.**
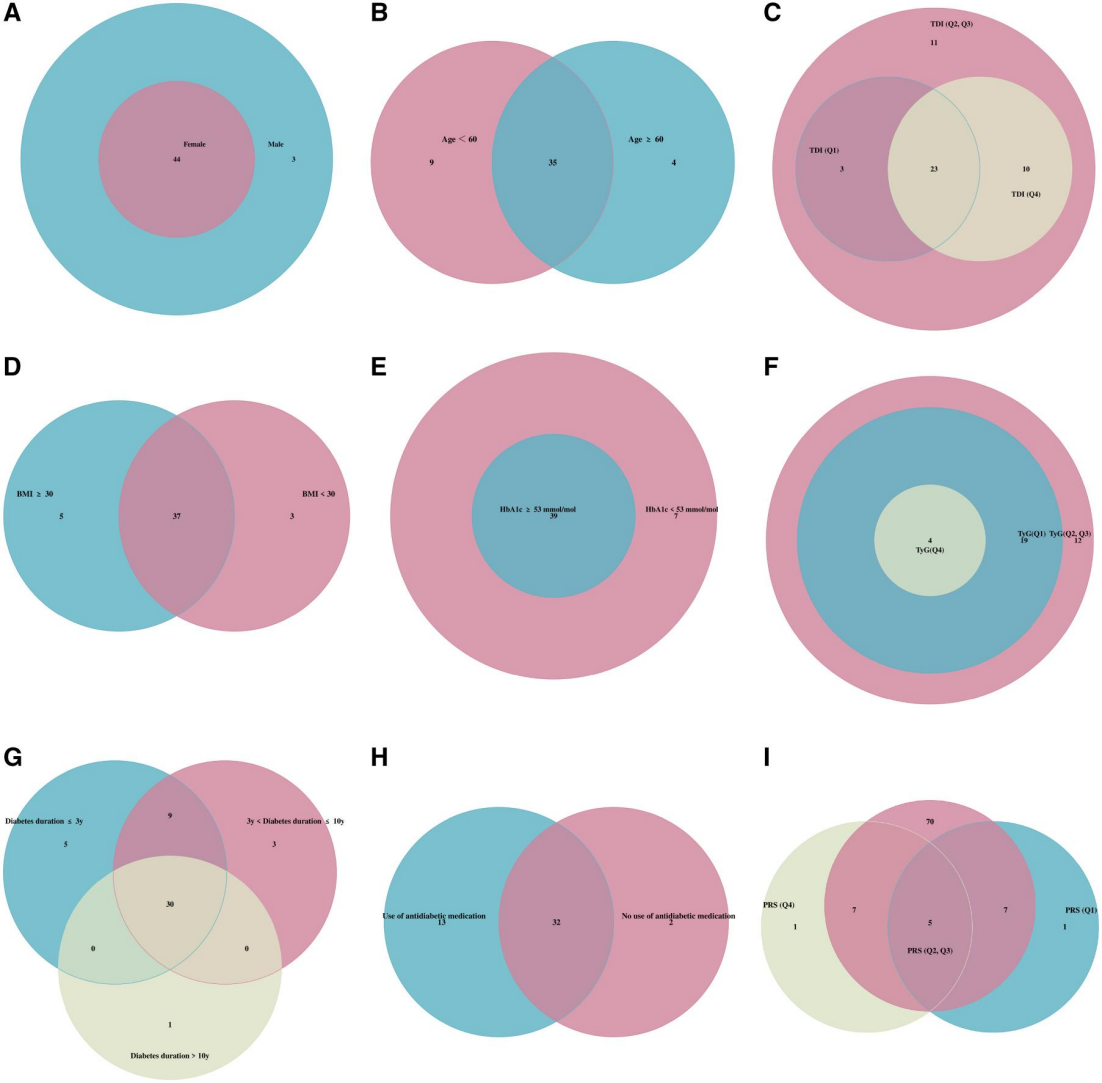
Venn diagram of mediating metabolic biomarkers in different subgroups. Number of metabolic biomarkers with mediating proportion ≥10% and overlap across groups are shown in different colors. (**A**) Sex (male or female). (**B**) Age (≥60 or <60 years). (**C**) TDI (low: Q1; medium: Q2 and Q3; high: Q4). (**D**) BMI (≥30 or <30). (**E**) HbA1c (≥53 or <53 mmol/mol). (**F**) TyG index (low: Q1; medium: Q2 and Q3; high: Q4). (**G**) Diabetes duration (≤3 years, >3 and ≤10 years, >10 years). (**H**) Use of antidiabetic medication. (**I**) Polygenic risk score (PRS) (low: Q1; medium: Q2 and Q3; high: Q4).

Subgroup analysis showed similar mediators across sex, age, and BMI, but more biomarkers mediated associations in participants with shorter diabetes duration, lower HbA1c, higher TDI, lower TyG index, antidiabetic medication use and medium DKD genetic risk (Figure 3; Supplementary Tables S10–S13). For CKD stages, most biomarkers mediated associations with stages 3–5, but none reached ≥10% for stages 1–2 or end-stage CKD (Supplementary Figure S11 and Tables S14–16).

Including metabolic signatures as covariates showed that low-risk smoking and moderate alcohol intake remained protective against DKD, whereas associations for diet, sleep and physical activity largely depended on metabolic pathways (Supplementary Figures S12 and S13).

### The genetic determinants of the mediating metabolic biomarkers

**Figure 4.**
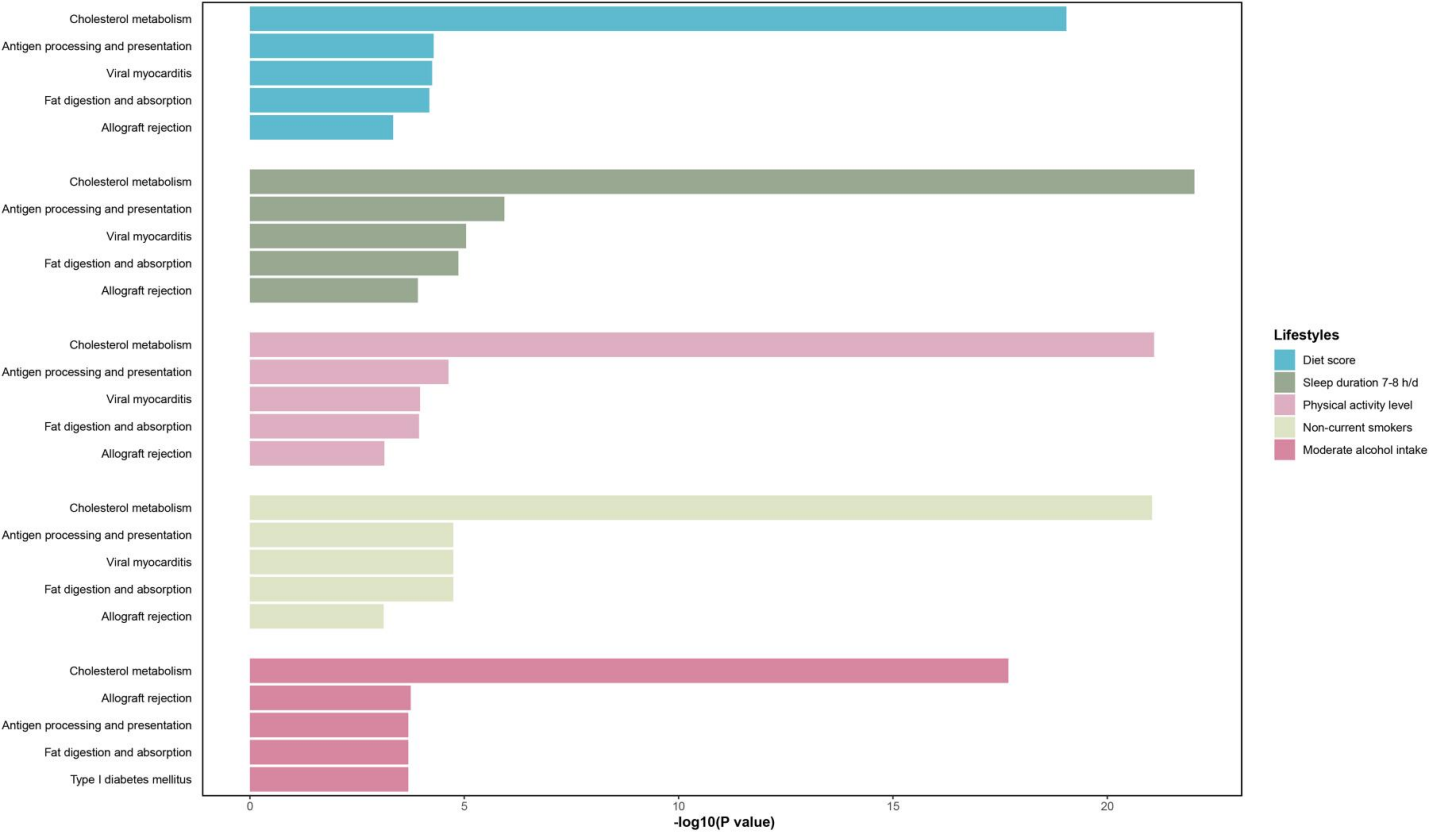
Gene enrichment analysis based on KEGG 2021 Human database. This figure shows the KEGG pathway enrichment results for genes associated with metabolic biomarkers mediating the relationships between lifestyle factors and DKD, identified via MAGMA gene-set analysis. For each lifestyle factor (diet, sleep, physical activity, smoking and alcohol), only the top five most significantly enriched pathways are displayed, ranked by *P* values. The length of each bar represents the statistical significance of enrichment (−log10[*P* values]).

Genetic analysis of mediating metabolic biomarkers identified 1418, 1248, 1486, 1491 and 596 genes related to diet, sleep, physical activity, smoking and alcohol intake, respectively (Supplementary Table S17). Across all lifestyle factors, genes were consistently enriched in lipid metabolism, particularly cholesterol, triglyceride, and sterol homeostasis (Figure 4; Supplementary Figures S14–21), as well as lipoprotein particle remodeling (HDL, chylomicron) and immune response pathways (MHC protein complexes, antigen processing). Unique enrichments included cholesterol transfer in alcohol intake, MHC Class II receptor activity in smoking and physical activity, fat digestion/absorption in alcohol, and viral myocarditis in smoking. Reactome/Wikipathway analysis highlighted plasma lipoprotein clearance in sleep and familial hyperlipidemia in alcohol intake and smoking. These genes were particularly enriched in liver tissues (Supplementary Figure S22 and Table S18).

In MR analysis, 25 metabolic biomarkers were significantly associated with DKD (Supplementary Figure S23 and Tables S19 and 20). Phospholipids to total lipids in medium HDL percentage showed the strongest positive association (HR = 1.28; 95% CI, 1.10–1.47; *P* = 1.12E−3), while cholesterol to total lipids in medium HDL percentage showed the strongest negative association (HR = 0.75; 95% CI, 0.65–0.87; *P* < 0.001), consistent with observational analysis. Reverse MR indicated DKD was associated with 13 metabolic biomarkers (Supplementary Figure S24 and Tables S21 and S22), with acetoacetate and phospholipids to total lipids in medium HDL showing bidirectional effects. Sensitivity analyses for heterogeneity and pleiotropy are shown in Supplementary Tables S23–S30.

## Discussion

This study employed UK Biobank data to investigate the associations between lifestyle factors, metabolic biomarkers, and DKD, as well as the mediation effects of metabolic biomarkers. MR analysis was used to mitigate potential confounders, and enrichment analysis mapped the identified mediating metabolic biomarkers to specific biological pathways, offering insights into the underlying mechanisms of DKD.

Previous studies have examined the impact of lifestyle habits on DKD through cohorts or clinical trials,^21–24^ but few have investigated the mediating mechanisms. Metabolomics studies in diabetes have largely focused on identifying disease-associated metabolites or building predictive models, highlighting branched-chain amino acids, acylcarnitines, and specific lipid species as predictors of type 2 diabetes,^25^ gestational diabetes,^26^ progression from prediabetes to diabetes,^27^ and microvascular^28^ and macrovascular complications.^29^ MR^30^ and network analysis^31^ have explored causal relationships and metabolic networks, but most studies treat metabolites as markers rather than mediators linking lifestyle to disease. Our metabolomics analysis provides greater resolution, detailing lipoprotein sizes, lipid distributions, and fatty acid profiles, enabling mechanistic insights. We systematically link lifestyle factors, including diet, physical activity, smoking and alcohol, to metabolic alterations and diabetes risk, highlighting modifiable pathways. Pathway enrichment confirmed known lipid and immune pathways, such as MHC Class II,^32^ and revealed novel associations, including plasma lipoprotein clearance and viral myocarditis. Our study advances previous work by focusing on metabolic mediation, evaluating multiple lifestyle dimensions simultaneously,^33^^,^^34^ and incorporating mediating pathways to develop predictive potential for mechanistic understanding and intervention strategies.^27^^,^^31^ Notably, our analysis identified several potentially novel mechanistic pathways linking lifestyle factors to DKD. For example, smoking may influence DKD risk via the “viral myocarditis” pathway, while sleep patterns may modulate risk through the “plasma lipoprotein clearance” pathway. In addition, pathways such as lipoprotein remodeling and MHC Class II involvement appear mechanistically relevant to DKD development. At the metabolite level, acetoacetate and the proportion of phospholipids in medium HDL showed bidirectional associations with DKD, highlighting specific, modifiable metabolic targets for intervention.

In diabetes and its complications, abnormal metabolite changes reflect core mechanisms of systemic metabolic dysregulation. Altered HDL-associated lipids affect cholesterol metabolism and are linked to DKD and cardiovascular complications. BCAA accumulation exacerbates insulin resistance via pathways such as mTOR, further promoting renal and cardiovascular risk. Metabolomics studies provide insights for early risk identification and targeted intervention.^13^^,^^14^ Our study suggests that N-isovaleroylglycine and valine contribute to diabetes complications through BCAA dysregulation, mitochondrial dysfunction, and systemic inflammation. Both are produced via valine catabolism (valine to isovalerate to N-isovalerylglycine) and have been linked to mitochondrial and glycine metabolism abnormalities in DKD.^13^^,^^14^ Dysregulated BCAA metabolism is associated with insulin resistance, type 2 diabetes, and microvascular complications, and elevated valine metabolites correlate with adipose dysfunction and hyperglycemia progression.^35–38^ N-isovaleroylglycine accumulation reflects mitochondrial stress, as BCAA intermediates impair ATP production, increase ROS, and disrupt calcium homeostasis, affecting nerves, repair cells, and podocytes.^38^^,^^39^ Ischemia/reperfusion models confirm that mitochondrial blockade and succinate accumulation induce oxidative stress,^40^ and elevated N-isovaleroylglycine may inhibit the urea cycle and disturb mitochondrial dynamics.^39^^,^^41^ Additionally, BCAAs interact with inflammatory and insulin signaling pathways, promoting low-grade inflammation that exacerbates ischemia and impairs wound healing, while valine metabolites modulate gut immunity and pro-inflammatory responses via short-chain fatty acid signaling and epigenetic regulation.^42–46^

In DKD, HDL-C decreases, triglycerides increase, and HDL function and miRNA regulation are impaired, contributing to microvascular damage.^47–55^ Triglyceride accumulation in the renal cortex and altered glycoprotein acetylation (GlycA) glycosylation exacerbate inflammation, fibrosis, and endothelial dysfunction.^56–68^ Acetoacetate and medium-sized HDL phospholipids exhibit bidirectional regulation in DKD. Ketones modulate renal energy metabolism, mitochondrial function, oxidative stress, and inflammation, while renal dysfunction alters their circulating levels.^69–91^ HDL phospholipids regulate anti-inflammatory, antioxidant, and cholesterol transport functions, but chronic inflammation remodels HDL and impairs its function.^92–101^ These interactions highlight metabolic-lipid networks influencing DKD progression and potential intervention targets.

Smoking alters sphingolipid and kynurenine metabolism, linking metabolic-immune networks to DKD.^11^^,^^102–108^ Immune and apoptosis signals intersect viral myocarditis pathways, suggesting involvement in DKD pathology.^109–111^ Sleep disruption affects lipid and energy metabolism via APOE, LPL, CETP, and LDLR, influencing DKD risk.^9^^,^^112–121^ Lipoprotein remodeling promotes renal injury through lipotoxicity, mitochondrial dysfunction, and inflammation, highlighting HDL transport as a potential intervention target.^58^^,^^88^^,^^122–134^ MHC II pathways indicate local antigen presentation and chronic inflammation may drive fibrosis, offering immunomodulatory therapeutic potential.^135^

More metabolic biomarkers mediate associations between lifestyle factors and advanced CKD (stages 3–5), consistent with prior findings linking phospholipids, glycolipids, and amino acids to CKD progression.^136–138^ Differences in mediation may reflect case numbers, and larger cohorts are needed for validation. Interactions with blood glucose suggest that better glycemic control may mitigate lifestyle-induced kidney metabolic disorders, warranting large clinical trials to assess its effect on kidney injury risk.

The predictive model and associated metabolite biomarkers developed in this study show promising clinical potential, though their broader implementation requires careful evaluation. Lipid and amino acid metabolites can be efficiently quantified using NMR spectroscopy platforms, such as Nightingale, which have been successfully applied in large cohorts like the UK Biobank,^20^ and assay costs are gradually decreasing with standardized protocols.^139^ Some key biomarkers, including HDL subclasses and GlycA, can also be measured using alternative immunoassays or biochemical methods, providing potential avenues for low-cost, high-throughput clinical testing.^140–142^ These metabolite biomarkers may be particularly suitable as complements to existing clinical measures, such as eGFR and urinary albumin, to facilitate refined risk stratification and early intervention in high-risk populations.^143–145^

Currently, DKD risk assessment primarily relies on eGFR and urinary albumin/protein levels, as recommended by KDIGO guidelines, which have limited sensitivity for early metabolic changes.^146^ In this study, the metabolite biomarkers we identified, including alterations in lipid and amino acid profiles, may serve as complementary indicators for early identification of high-risk individuals and for guiding follow-up and intervention timing. Using NMR and targeted mass spectrometry platforms, these measurements show high reproducibility and quantifiability in large cohorts, highlighting their potential clinical translational value.^146^ Compared with conventional clinical tools, such as Wagner grading and neuropathy scores, metabolite features can reveal molecular links between systemic metabolic dysregulation and microvascular injury, addressing limitations of morphology- and symptom-based assessments. Specifically, NMR-detectable GlycA reflects systemic low-grade inflammation and has been associated with early kidney injury and diabetes-related inflammatory pathways, suggesting its potential for early detection of microvascular complications, particularly DKD.^147^^,^^148^ Additionally, studies of lipid and lipoprotein profiles indicate that different HDL/LDL subclasses and particle concentrations are significantly associated with neuropathy and nephropathy,^149^^,^^150^ suggesting that lipid metabolic patterns may complement clinical grading in assessing complication type and severity.

Lifestyle interventions may help delay DKD onset and progression by modulating metabolic pathways. Dyslipidemia, particularly low HDL-C and high triglycerides, and TyG are associated with DKD risk and progression,^151^^,^^152^ suggesting that targeting lipid and glucolipid metabolism could confer renal protection. Studies indicate that the Mediterranean diet and ω-3 supplementation improve HDL lipidomic profiles,^153^ while diet and exercise reduce GlycA levels and systemic inflammation,^154^ supporting the mechanistic feasibility of lifestyle optimization for delaying DKD progression.

Our study has several strengths. The UK Biobank NMR metabolomics dataset enables precise profiling of metabolic biomarkers across key pathways. Longitudinal follow-up cohort design and MR analysis confirm causal links between metabolic biomarkers and DKD, eliminating confounding factors. However, limitations exist. The study was conducted in a single center, and external validation in multi-center or larger cohorts is needed to confirm the reproducibility and applicability of our findings. DKD incidence was relatively low, potentially limiting statistical power in some subgroups. The relatively small sample size may also affect the robustness of our predictive model, making it more susceptible to overfitting and reducing its generalizability to broader populations. Moreover, although our study included follow-up, the limited number of sampling time points and relatively short follow-up duration restrict our ability to fully assess the predictive capacity of the identified biomarkers and mediation pathways. Identifying DKD from hospitalization records and death registries may lead to underreporting due to incomplete primary care data. Future studies incorporating multi-center data, larger sample sizes, and repeated measurements over time are warranted to validate and extend our findings.

## Conclusions

Our findings demonstrate that lifestyle factors significantly influence the metabolome and may modulate DKD progression through various metabolic pathways. Future research should focus on validating these results with independent, diverse cohorts, incorporating longitudinal data to capture dynamic changes in metabolic biomarkers, and exploring lifestyle-specific interventions for DKD.

## Supplementary Material

hcaf281_Supplementary_Data

## Data Availability

Data generated and analyzed in this study are available from corresponding author upon request except for UKB data, which only available directly on application.
