# Supplementary material for: Lifestyles, metabolome and diabetic kidney disease: a cohort study
Source: QJM. 2025 Nov 17;119(4):290–303. doi: 10.1093/qjmed/hcaf281 (PMC13134830; doi:10.1093/qjmed/hcaf281)
Supplement: hcaf281_Supplementary_Data [file hcaf281_supplementary_data.zip › hcaf281_Supplementary_Data/Supplementary_Materials-clean_copy.pdf]

Supplementary Materials

Supplementary Methods 3
Study Population 3
Lifestyle Exposures 3
Metabolic Biomarkers 3
Ascertainment of Outcomes 4
Ascertainment of Covariates 4
Subgroup Analysis 4
Identification of Metabolic Signature Reflecting Lifestyles 4
Mendelian Randomization 5
Statistical Analysis 5
Supplementary Tables – available as an excel file 9
Supplementary Figures 11
Figure S1: Associations between lifestyles and DKD incidence in observational analysis 11
Figure S2: Associations between lifestyles and eGFR in observational analysis 12
Figure S3: Associations between lifestyles and DKD mortality in observational analysis 13
Figure S4: Associations between metabolic biomarkers and DKD mortality in observational analysis 14
Figure S5: Associations between metabolic biomarkers and eGFR in observational analysis 15
Figure S6: Associations between lifestyles and metabolic biomarkers in model 2 of observational analysis 16
Figure S7: Associations between lifestyles and metabolic biomarkers in model 3 of observational analysis 17
Figure S8: Interaction effects between metabolic biomarkers and genetic and environmental factors in model 3 of observational analysis 18
Figure S9: Clustering analysis based on PCA dimensionality reduction 19
Figure S10: Clustering analysis based on OPLS-DA dimensionality reduction 20
Figure S11: Venn diagram of mediating metabolic biomarkers for incidence of DKD progressing to different CKD stages 21
Figure S12: Associations between lifestyles and DKD incidence independent of metabolic signatures in observational analysis 22
Figure S13: Associations between lifestyles and DKD mortality independent of metabolic signatures in observational analysis 23
Figure S14: Venn diagram of enriched pathways for lifestyle-associated metabolic biomarkers 24
Figure S15: Gene enrichment analysis based on BioPlanet 2019 database 26
Figure S16: Gene enrichment analysis based on MsigDB Hallmark 2020 database 27
Figure S17: Gene enrichment analysis based on Reactome 2022 database 28
Figure S18: Gene enrichment analysis based on WikiPathways 2024 Human database 29
Figure S19: Gene enrichment analysis based on GO Biological Process 2023 database 30

Figure S20: Gene enrichment analysis based on GO Cellular Component 2023 database 31
Figure S21: Gene enrichment analysis based on GO Molecular Function 2023 database 32
Figure S22: Gene enrichment analysis based on GTEx Tissues V8 2023 database........33
Figure S23: Mendelian randomization analysis with metabolic biomarkers as exposures and DKD or eGFR as outcomes...34
Figure S24: Mendelian randomization analysis with DKD or eGFR as exposures and metabolic biomarkers as outcomes 35

Supplementary Methods

Study Population

The study design was shown in **Supplementary Figure 1**. The UK Biobank (UKB) is a large-scale, longitudinal cohort study that enrolled participants aged 40 to 70 from the general population between 2006 and 2010 across 22 centers in the United Kingdom. Ethical approval was obtained from the North West Multicenter Research Ethics Committee, and all participants provided written informed consent.(1) The study was conducted under UKB application number 105945. Inclusion criteria for participants were as follows: presence of diabetes at baseline (defined using International Classification of Diseases, Tenth Revision codes E11, E12, E13, and E14, as well as self-reported medical conditions), absence of DKD at baseline, and availability of complete lifestyle and covariate data (**Supplementary Table 1**).

Lifestyle Exposures

Five lifestyle factors, including diet, sleep duration, physical activity, smoking status, and alcohol intake, were assessed in this study. Diet was evaluated using a healthy diet score.(2) Healthy sleep duration was defined as 7 to 8 hours per day.(3) Physical activity levels were classified into four categories: no activity, low activity, moderate activity, and high activity.(4) Smoking status was self-reported, with non-current smokers categorized as low-risk.(5) Moderate alcohol intake was defined as drinking once or twice a week, classified as low-risk drinking status (**Supplementary Table 1**).(6, 7)

Metabolic Biomarkers

Metabolic biomarker data from the UK Biobank, collected between March 2006 and October 2010 using NMR spectroscopy on the Nightingale platform, were utilized in this study.(8, 9) The platform implemented strict quality control (QC) measures during each 96-well plate assay to ensure data stability and reproducibility. Each plate included two internal control samples provided by Nightingale Health to monitor measurement consistency across different NMR spectrometers and assay batches, as well as two blinded repeat samples from UK Biobank (aliquots of the same participant distributed in different wells, unblinded post-analysis) to assess technical reproducibility throughout the sample processing and measurement workflow. QC results were expressed as coefficients of variation (CV), with most metabolic biomarkers showing CVs below 5% in the combined Phase 1 and Phase 2 datasets, indicating high measurement repeatability. Following data acquisition, all spectra underwent automated quality checks, and metabolite quantification was performed using Nightingale Health proprietary software (2020 biomarker quantification library) with standardization. Although biomarker distributions across spectrometers were largely consistent, investigators could adjust for the “spectrometer” variable in epidemiological analyses; for instance, in the Phase 2 release, Nightingale Health provided a corrected variable for alanine, the biomarker most sensitive to instrument variation, reflecting proactive management of technical variability. Previous studies have also discussed approaches to mitigate technical variation in UK Biobank NMR data. Additionally, the platform established a sample quality annotation system to identify degradation or contamination issues, at both the sample and biomarker level. When a metabolite carries a quality annotation but retains a quantitative value, it indicates minimal interference and reliable measurements. Researchers can use

these annotations for sensitivity analyses, such as excluding samples labeled “low protein,” to control for potential dilution effects and enhance analytical robustness.

Participants with complete metabolomic data of 251 metabolic biomarkers (n = 9,412) were included for analysis involving metabolic biomarkers. Abbreviations and full names of metabolic biomarkers were in **Supplementary Table 2.** Inverse normal rank transformation was applied to each metabolite to account for batch effects and to normalize the distributions for subsequent analyses.

Ascertainment of Outcomes

DKD was identified using the International Classification of Diseases, Tenth Revision (ICD-10) codes E11.2, E12.2, E13.2, E14.2, N18.0, N18.1, N18.2, N18.3, N18.4, N18.5, N18.8, and N18.9.(5) To assess the effects of lifestyles and metabolic biomarkers on DKD of different severities, individuals who ultimately progress to chronic kidney disease (CKD) are classified into six stages using ICD-10 (stage 1: N18.1; stage 2: N18.2; stage 3: N18.3; stage 4: N18.4; stage 5: N18.5; end-stage: N18.0). DKD mortality was derived from the cause of death based on ICD-10 mentioned above, using data provided by patients and collected by the NHS as part of their care and support (**Supplementary Table 1**). Participants were followed from the date of recruitment until the occurrence of DKD diagnosis, death, or the last follow-up date (September 31, 2021), whichever came first.

Ascertainment of Covariates

Model 1 included age at recruitment, sex, Townsend deprivation index (TDI), ethnicity, and education level (college or university degree). Model 2 additionally incorporated body mass index (BMI), glycated hemoglobin (HbA1c), diabetes duration, hypertension prevalence, use of antidiabetic medications, use of antihypertensive medications, use of lipid-lowering medications, and triglyceride-glucose (TyG) index (**Supplementary Table 1**). Model 3 included same covariates as model 2, but excluded participants with estimated glomerular filtration rate (eGFR) < 60.

Subgroup Analysis

To assess the interactions between metabolic biomarkers and sociodemographic factors and blood glucose control, association and mediation analysis were conducted based on population stratified by sex (male or female), age (≥60 years or <60 years), TDI (low: Q1; medium: Q2 and Q3; high: Q4), BMI (≥30 or <30), HbA1c (≥53 mmol/mol or <53 mmol/mol), use of antidiabetic medication, diabetes duration (≤3 years, >3 and ≤10 years, >10 years), TyG index (low: Q1; medium: Q2 and Q3; high: Q4). Details of variables above were in **Supplementary Table 1**.

Identification of Metabolic Signature Reflecting Lifestyles

Baseline metabolomic profiles from the UK Biobank were used to investigate the metabolic signatures associated with each lifestyle factor. Elastic net regression was applied to examine the relationships between 251 metabolic biomarkers and five lifestyle variables, based on glmnet R package version 4.1-8. This approach combines the regularization techniques of Lasso and ridge regression. The metabolic signatures were calculated as the weighted sum of the selected metabolic biomarkers.

Mendelian Randomization

GWAS summaries of metabolic biomarkers were downloaded from IEU OpenGWAS project (https://gwas.mrcieu.ac.uk/datasets/?gwas_id__icontains=met-d),(10) the summaries of DKD and eGFR in diabetes population were from study of Zuydam et al. (2018). (11) To explicitly address the three core assumptions of Mendelian randomization (MR)–relevance, independence, and exclusion restriction–we applied the following strategies. For relevance, instrumental variants (IVs) were required to be significantly associated with the exposure. Specifically, we selected SNPs with $P < 1\times10^{-5}$ and effect allele frequency (EAF) > 0.01, and excluded weak instruments with F statistic ≤ 10 to ensure sufficient IV strength. For independence, IVs were required to be independent of potential confounders of the exposure-outcome relationship. To minimize confounding, IVs were clumped at $r^2 = 0.01$ to retain only independent SNPs, and palindromic variants were removed. For exclusion restriction, IVs were assumed to influence the outcome solely through the exposure and not via alternative pathways. This assumption was assessed using MR-Egger intercept tests and MR-PRESSO global tests to detect horizontal pleiotropy. Causal effects were primarily estimated using inverse variance weighted (IVW) with a random-effects model. Additional MR methods, including weighted median, penalized weighted median, IVW radial, and maximum likelihood, were employed to provide robust estimates and validate findings. Sensitivity analyses included pleiotropy assessment via MR-Egger intercept P values and MR-PRESSO, heterogeneity evaluation using Cochran's Q statistic, and reverse MR analysis with Steiger filtering to confirm causal direction. Analyses were conducted using the TwoSampleMR R package (v0.5.10).

Statistical Analysis

Continuous baseline characteristics were compared using t-tests, while categorical variables were analyzed with the chi-squared test or Fisher's exact test. Cox proportional hazards models were employed to assess the associations between lifestyle factors and DKD, adjusting for covariate models 1 and 2. To evaluate the effects of lifestyles on DKD independently of metabolic biomarkers, metabolic signatures corresponding to each lifestyle factor were additionally adjusted for. Associations between lifestyles and eGFR were examined using general linear models (GLM) to test robustness. GLM was also used to test associations between lifestyles and metabolic biomarkers in both models 1 and 2. Associations between metabolic biomarkers and DKD were assessed using both Cox proportional hazards models and MR models. In MR analysis, significant associations were defined as those with a P value < 0.05, based on inverse-variance weighted multiplicative random effects. Heterogeneity, pleiotropy, and reverse causality were evaluated in the MR analysis to account for potential confounders. The effects of metabolic biomarkers on eGFR were further analyzed using GLM and MR models to identify potential causal relationships. Mediation analysis was performed using the mediation R package version 4.5.0, focusing on metabolic biomarkers significantly associated with both lifestyles and DKD. To test sensitivity, observational analysis was repeated in participants with eGFR ≥ 60 (model 3). eGFR was calculated using serum creatinine levels based on the 2021 race-free Chronic Kidney Disease Epidemiology Collaboration (CKD-EPI) equation.(12) Observational analysis above were conducted in each subgroup using model 1. We performed PCA and OPLS-DA for dimensionality reduction and clustering of metabolites,(13-15) using DKD status and subgroup classifications as grouping variables to visualize clustering results. To further investigate the underlying biological mechanisms, genome-wide association study (GWAS) summary statistics for these

mediating metabolic biomarkers were downloaded from IEU OpenGWAS project (https://gwas.mrcieu.ac.uk/datasets/?gwas_id__icontains=met-d),(10) and analyzed using Multi-marker Analysis of Genomic Annotation (MAGMA) .(16) Enrichment analysis was conducted on genes corresponding to these mediating metabolic biomarkers for each lifestyle factor, utilizing BioPlanet,(17) KEGG,(18) MSigDB,(19) Reactome,(20) Wikipathways,(21) and Gene Ontology (GO, including biological process, cellular component, and molecular function).(22) Tissue-specific gene expression data were annotated using GTEx v8 .(23) False discovery rate correction was applied for multiple comparisons. Statistical analyses were performed using R version 4.3.1.

References

1. Backman JD, Li AH, Marcketta A, Sun D, Mbatchou J, Kessler MD, et al. Exome sequencing and analysis of 454,787 UK Biobank participants. Nature. 2021;599(7886):628-34.
2. Wang M, Zhou T, Song Y, Li X, Ma H, Hu Y, et al. Joint exposure to various ambient air pollutants and incident heart failure: a prospective analysis in UK Biobank. European heart journal. 2021;42(16):1582-91.
3. Fan M, Sun D, Zhou T, Heianza Y, Lv J, Li L, et al. Sleep patterns, genetic susceptibility, and incident cardiovascular disease: a prospective study of 385 292 UK biobank participants. European heart journal. 2020;41(11):1182-9.
4. Hanlon P, Nicholl BI, Jani BD, Lee D, McQueenie R, Mair FS. Frailty and pre-frailty in middle-aged and older adults and its association with multimorbidity and mortality: a prospective analysis of 493 737 UK Biobank participants. The Lancet Public health. 2018;3(7):e323-e32.
5. Geng T, Zhu K, Lu Q, Wan Z, Chen X, Liu L, et al. Healthy lifestyle behaviors, mediating biomarkers, and risk of microvascular complications among individuals with type 2 diabetes: A cohort study. PLoS medicine. 2023;20(1):e1004135.
6. Elovainio M, Komulainen K, Sipilä PN, Pulkki-Råback L, Cachón Alonso L, Pentti J, et al. Association of social isolation and loneliness with risk of incident hospital-treated infections: an analysis of data from the UK Biobank and Finnish Health and Social Support studies. The Lancet Public health. 2023;8(2):e109-e18.
7. Rosoff DB, Davey Smith G, Mehta N, Clarke TK, Lohoff FW. Evaluating the relationship between alcohol consumption, tobacco use, and cardiovascular disease: A multivariable Mendelian randomization study. PLoS medicine. 2020;17(12):e1003410.
8. Julkunen H, Cichońska A, Slagboom PE, Würtz P. Metabolic biomarker profiling for identification of susceptibility to severe pneumonia and COVID-19 in the general population. eLife. 2021;10.
9. Ritchie SC, Surendran P, Karthikeyan S, Lambert SA, Bolton T, Pennells L, et al. Quality control and removal of technical variation of NMR metabolic biomarker data in ~120,000 UK Biobank participants. Scientific data. 2023;10(1):64.
10. Julkunen H, Cichońska A, Tiainen M, Koskela H, Nybo K, Mäkelä V, et al. Atlas of plasma NMR biomarkers for health and disease in 118,461 individuals from the UK Biobank. Nature communications. 2023;14(1):604.
11. van Zuydam NR, Ahlqvist E, Sandholm N, Deshmukh H, Rayner NW, Abdalla M, et al. A Genome-Wide Association Study of Diabetic Kidney Disease in Subjects With Type 2 Diabetes. Diabetes. 2018;67(7):1414-27.
12. Inker LA, Eneanya ND, Coresh J, Tighiouart H, Wang D, Sang Y, et al. New Creatinine- and Cystatin C-Based Equations to Estimate GFR without Race. The New England journal of medicine. 2021;385(19):1737-49.
13. Jolliffe IT, Cadima J. Principal component analysis: a review and recent developments. Philosophical transactions Series A, Mathematical, physical, and engineering sciences. 2016;374(2065):20150202.
14. Worley B, Powers R. PCA as a practical indicator of OPLS-DA model reliability. Current Metabolomics. 2016;4(2):97-103.
15. Trygg J, Wold S. Orthogonal projections to latent structures (O-PLS). 2002;16(3):119-28.
16. de Leeuw CA, Mooij JM, Heskes T, Posthuma D. MAGMA: generalized gene-set analysis of GWAS data. PLoS computational biology. 2015;11(4):e1004219.

17. Huang R, Grishagin I, Wang Y, Zhao T, Greene J, Obenauer JC, et al. The NCATS BioPlanet - An Integrated Platform for Exploring the Universe of Cellular Signaling Pathways for Toxicology, Systems Biology, and Chemical Genomics. Frontiers in pharmacology. 2019;10:445.
18. Kanehisa M, Furumichi M, Sato Y, Matsuura Y, Ishiguro-Watanabe M. KEGG: biological systems database as a model of the real world. Nucleic acids research. 2024.
19. Castanza AS, Recla JM, Eby D, Thorvaldsdóttir H, Bult CJ, Mesirov JP. Extending support for mouse data in the Molecular Signatures Database (MSigDB). Nature methods. 2023;20(11):1619-20.
20. Milacic M, Beavers D, Conley P, Gong C, Gillespie M, Griss J, et al. The Reactome Pathway Knowledgebase 2024. Nucleic acids research. 2024;52(D1):D672-d8.
21. Agrawal A, Balcı H, Hanspers K, Coort SL, Martens M, Slenter DN, et al. WikiPathways 2024: next generation pathway database. Nucleic acids research. 2024;52(D1):D679-d89.
22. The Gene Ontology resource: enriching a GOld mine. Nucleic acids research. 2021;49(D1):D325-d34.
23. The Genotype-Tissue Expression (GTEx) project. Nature genetics. 2013;45(6):580-5.

Supplementary Tables – available as an excel file

Table S1: Field ID of phenotypes in UK Biobank
Table S2: Full names and abbreviations of metabolic biomarkers
Table S3: Baseline characteristics of DKD progressing to different CKD stages
Table S4: Associations between metabolic biomarkers and DKD incidence in observational analysis
Table S5: Associations between metabolic biomarkers and DKD mortality in observational analysis
Table S6: Associations between metabolic biomarkers and eGFR in observational analysis
Table S7: Associations between lifestyles and metabolic biomarkers in observational analysis
Table S8: Mediation effects of metabolic biomarkers between lifestyles and DKD incidence in observational analysis
Table S9: Mediation effects of metabolic biomarkers between lifestyles and DKD mortality in observational analysis
Table S10: Associations between lifestyles and DKD incidence in different subgroups
Table S11: Associations between lifestyles and metabolic biomarkers in different subgroups
Table S12: Associations between metabolic biomarkers and DKD incidence in different subgroups
Table S13: Mediation effects of metabolic biomarkers between lifestyles and DKD incidence in different subgroups
Table S14: Associations between lifestyles and incidence of DKD progressing to different stages of CKD
Table S15: Associations between metabolic biomarkers and incidence of DKD progressing to different stages of CKD
Table S16: Mediation effects of metabolic biomarkers between lifestyles and incidence of DKD progressing to different stages of CKD
Table S17: Genes corresponding to mediatory metabolic biomarkers of each lifestyle
Table S18: Gene enrichment analysis
Table S19: MR analysis with metabolic biomarkers as exposure and DKD as outcome
Table S20: MR analysis with metabolic biomarkers as exposure and eGFR as outcome
Table S21: MR analysis with DKD as exposure and metabolic biomarkers as outcome
Table S22: MR analysis with eGFR as exposure and metabolic biomarkers as outcome
Table S23: Heterogeneity analysis of MR with metabolic biomarkers as exposure and DKD as outcome
Table S24: Heterogeneity analysis of MR with metabolic biomarkers as exposure and eGFR as outcome
Table S25: Heterogeneity analysis of MR with DKD as exposure and metabolic biomarkers as outcome
Table S26: Heterogeneity analysis of MR with eGFR as exposure and metabolic biomarkers as outcome

Table S27: Pleiotropy analysis of MR with metabolic biomarkers as exposure and DKD as outcome

Table S28: Pleiotropy analysis of MR with metabolic biomarkers as exposure and eGFR as outcome

Table S29: Pleiotropy analysis of MR with DKD as exposure and metabolic biomarkers as outcome

Table S30: Pleiotropy analysis of MR with eGFR as exposure and metabolic biomarkers as outcome

Supplementary Figures

Figure S1: Associations between lifestyles and DKD incidence in observational analysis

Lifestyles	HR (95%CI)	P value
**Model 1**		
Diet score	0.94 (0.91−0.96)	8.6E−6
Sleep duration 7−8 h/d	0.84 (0.79−0.90)	1.3E−7
Physical activity level	0.76 (0.73−0.79)	1.8E−38
Non−current smokers	0.82 (0.74−0.91)	2.6E−4
Moderate alcohol intake	0.87 (0.80−0.94)	3.4E−4
**Model 2**		
Diet score	0.95 (0.93−0.98)	1.1E−3
Sleep duration 7−8 h/d	0.92 (0.86−0.98)	9.6E−3
Physical activity level	0.85 (0.81−0.89)	6.2E−14
Non−current smokers	0.78 (0.70−0.86)	2.1E−6
Moderate alcohol intake	0.88 (0.82−0.95)	1.9E−3
**Model 3**		
Diet score	0.97 (0.93−1.00)	4.0E−2
Sleep duration 7−8 h/d	0.90 (0.83−0.97)	4.4E−3
Physical activity level	0.87 (0.83−0.92)	5.2E−8
Non−current smokers	0.72 (0.64−0.81)	2.1E−8
Moderate alcohol intake	0.87 (0.80−0.96)	3.5E−3

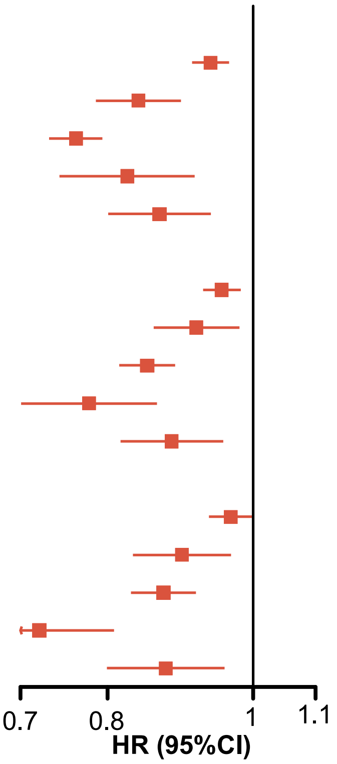

Results were presented as hazard ratio (HR) and 95%CI. Model 1 adjusted for age at recruitment, sex, Townsend deprivation index, ethnicity, and education level (college or university degree). Model 2 additionally adjusted body mass index (BMI), glycated hemoglobin (HbA1c), diabetes duration, hypertension prevalence, use of anti-hyperglycaemic medications, use of antihypertensive medications, and use of lipid-lowering medications. Model 3 included same covariates as model 2, but excluded participants with eGFR < 60.

Figure S2: Associations between lifestyles and eGFR in observational analysis

Lifestyles	Beta (95%CI)	P value
**Model 1**		
Diet score	0.69 (0.52−0.86)	2.1E-16
Sleep duration 7−8 h/d	0.50 (0.12−0.88)	9.8E-3
Physical activity level	0.95 (0.69−1.21)	6.8E-13
Non−current smokers	2.40 (1.79−3.01)	1.6E-14
Moderate alcohol intake	0.04 (−0.4−0.48)	8.7E-1
**Model 2**		
Diet score	0.68 (0.52−0.84)	1.9E-16
Sleep duration 7−8 h/d	0.21 (−0.17−0.59)	2.7E-1
Physical activity level	0.50 (0.24−0.76)	1.7E-4
Non−current smokers	2.06 (1.46−2.66)	1.8E-11
Moderate alcohol intake	−0.1 (−0.53−0.33)	6.5E-1

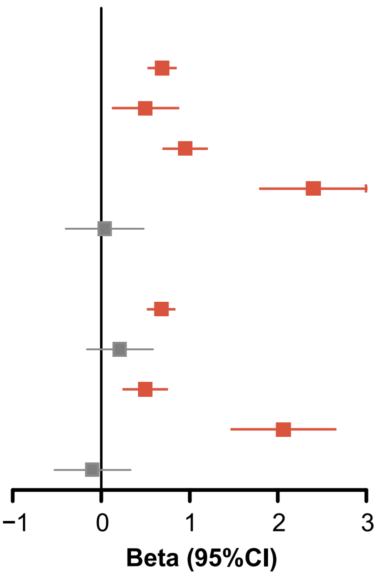

Results were presented as hazard ratio (HR) and 95%CI. Model 1 adjusted for age at recruitment, sex, Townsend deprivation index, ethnicity, and education level (college or university degree). Model 2 additionally adjusted body mass index (BMI), glycated haemoglobin (HbA1c), diabetes duration, hypertension prevalence, use of anti-hyperglycaemic medications, use of antihypertensive medications, and use of lipid-lowering medications.

Figure S3: Associations between lifestyles and DKD mortality in observational analysis

Lifestyles	HR (95%CI)	P value
**Model 1**		
Diet score	0.92 (0.85−1.00)	3.8E-2
Sleep duration 7−8 h/d	0.76 (0.64−0.91)	2.2E-3
Physical activity level	0.64 (0.58−0.71)	1.1E-17
Non−current smokers	0.68 (0.53−0.87)	2.6E-3
Moderate alcohol intake	0.88 (0.71−1.08)	2.2E-1
**Model 2**		
Diet score	0.95 (0.88−1.03)	1.9E-1
Sleep duration 7−8 h/d	0.88 (0.74−1.05)	1.7E-1
Physical activity level	0.77 (0.69−0.86)	1.2E-6
Non−current smokers	0.63 (0.49−0.81)	3.7E-4
Moderate alcohol intake	0.90 (0.73−1.12)	3.5E-1
**Model 3**		
Diet score	0.98 (0.89−1.09)	7.5E-1
Sleep duration 7−8 h/d	0.90 (0.72−1.14)	4.0E-1
Physical activity level	0.79 (0.69−0.91)	1.3E-3
Non−current smokers	0.48 (0.35−0.65)	3.7E-6
Moderate alcohol intake	0.76 (0.57−1.03)	7.4E-2

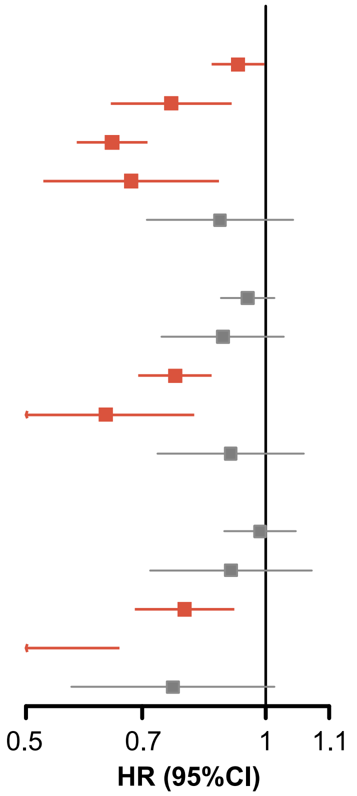

Results were presented as hazard ratio (HR) and 95%CI. Model 1 adjusted for age at recruitment, sex, Townsend deprivation index, ethnicity, and education level (college or university degree). Model 2 additionally adjusted body mass index (BMI), glycated haemoglobin (HbA1c), diabetes duration, hypertension prevalence, use of anti-hyperglycaemic medications, use of antihypertensive medications, and use of lipid-lowering medications. Model 3 included same covariates as model 2, but excluded participants with eGFR < 60.

Figure S4: Associations between metabolic biomarkers and DKD mortality in observational analysis

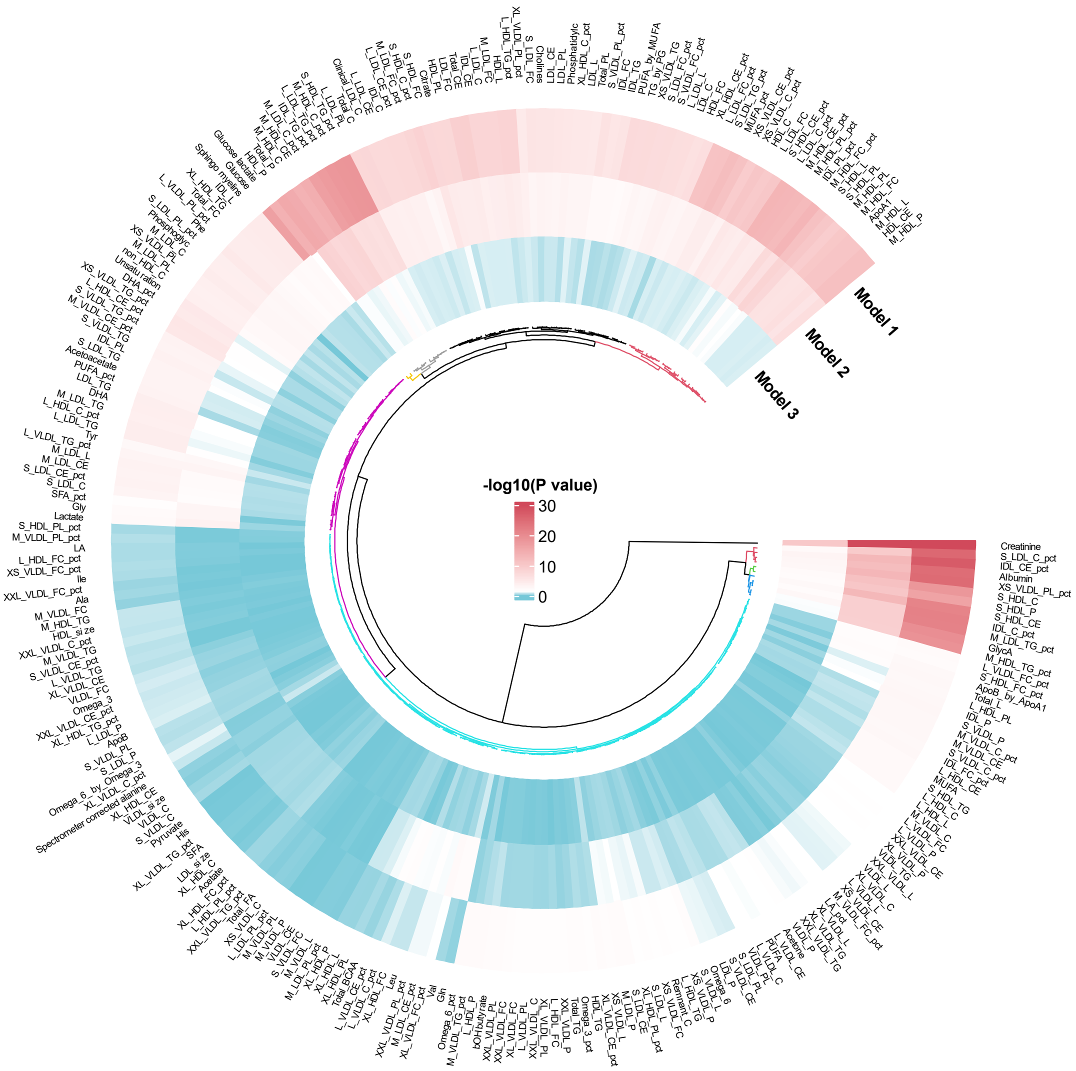

Results based on model 1, 2 and 3 were presented in heatmap. The red colour indicates statistical significance, while blue represents non-significance. The intensity of the colour corresponds to the magnitude of the p-value. Clustering patterns are presented using a dendrogram.

Figure S5: Associations between metabolic biomarkers and eGFR in observational analysis

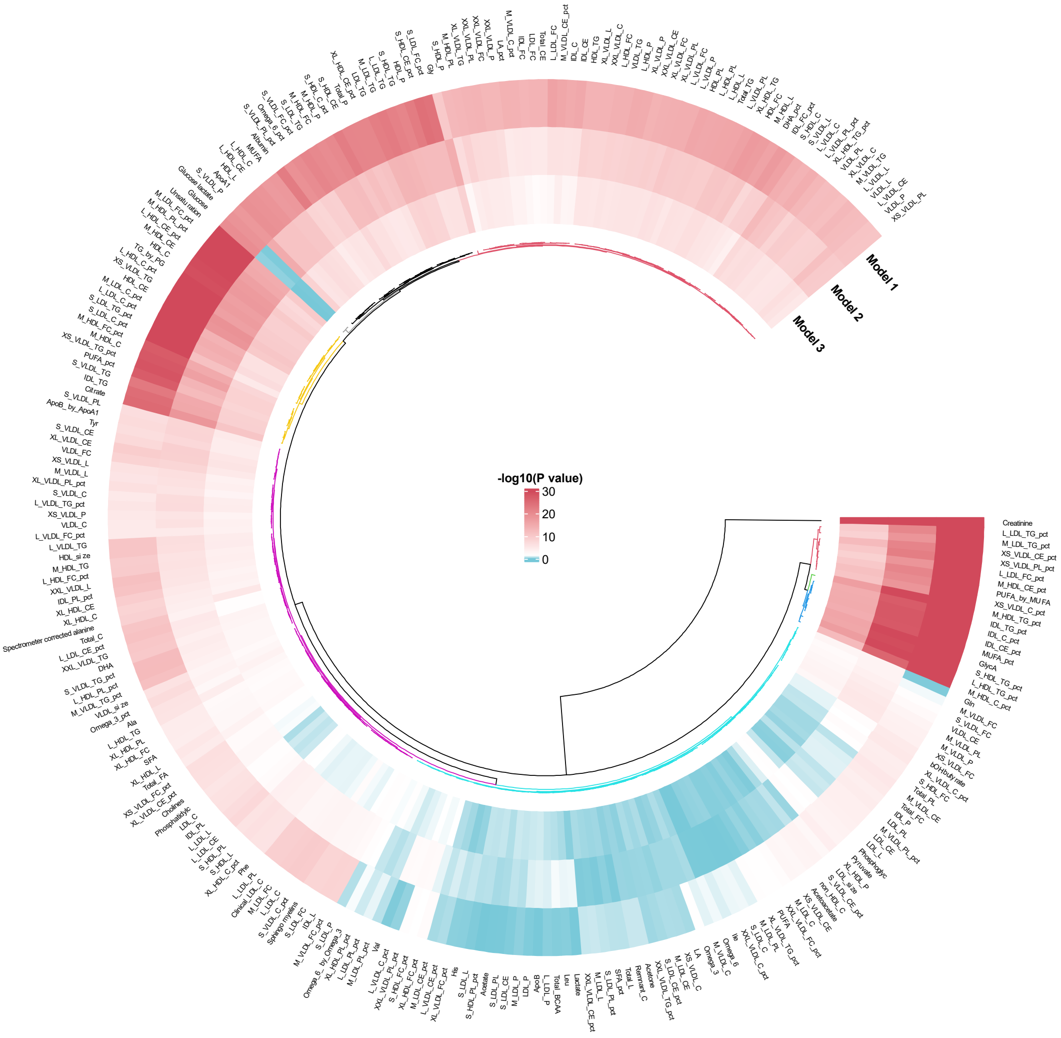

Results based on model 1, 2 and 3 were presented in heatmap. The red colour indicates statistical significance, while blue represents non-significance. The intensity of the colour corresponds to the magnitude of the p-value. Clustering patterns are presented using a dendrogram.

Figure S6: Associations between lifestyles and metabolic biomarkers in model 2 of observational analysis

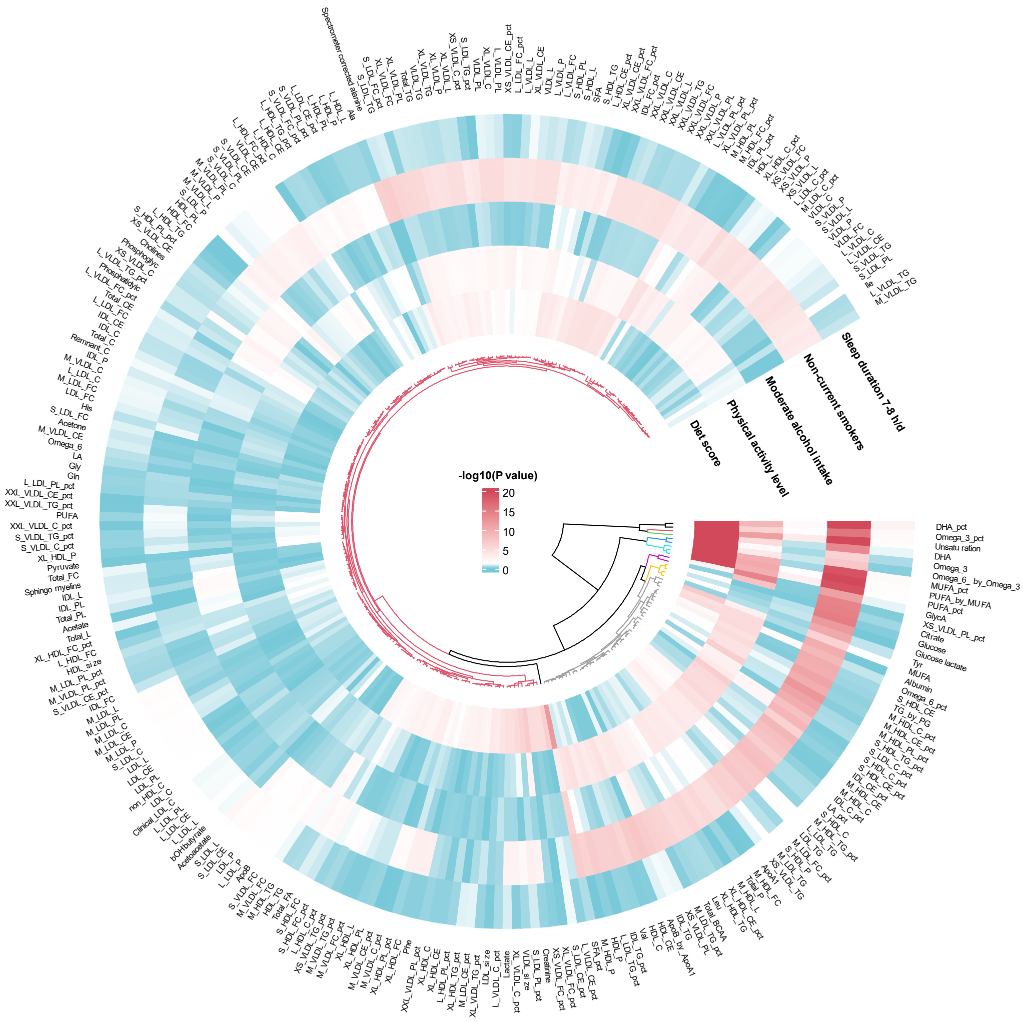

Results based on model 2 was presented in heatmap. The red colour indicates statistical significance, while blue represents non-significance. The intensity of the colour corresponds to the magnitude of the p-value. Clustering patterns are presented using a dendrogram.

Figure S7: Associations between lifestyles and metabolic biomarkers in model 3 of observational analysis

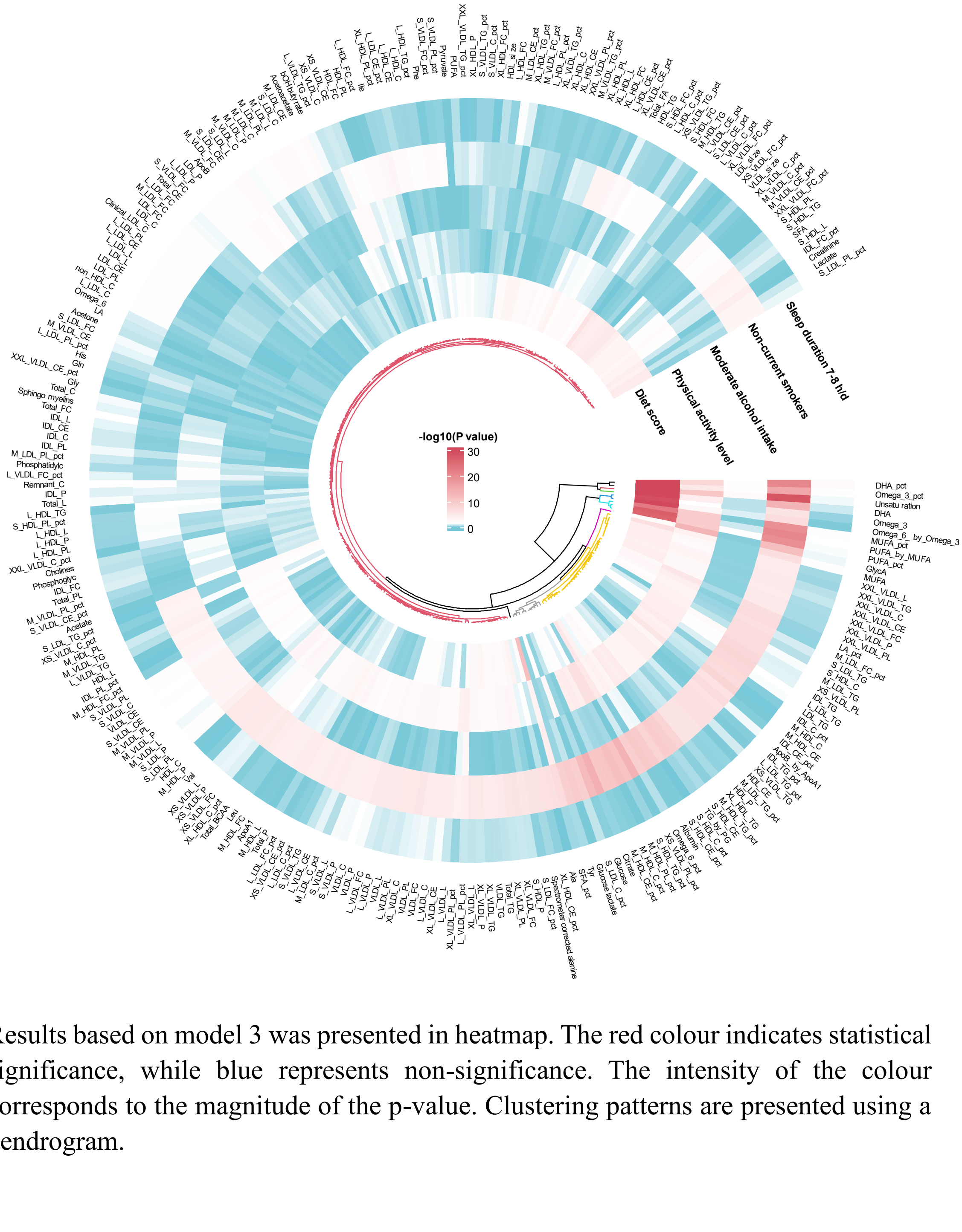

Results based on model 3 was presented in heatmap. The red colour indicates statistical significance, while blue represents non-significance. The intensity of the colour corresponds to the magnitude of the p-value. Clustering patterns are presented using a dendrogram.

Figure S8: Interaction effects between metabolic biomarkers and genetic and environmental factors in model 3 of observational analysis

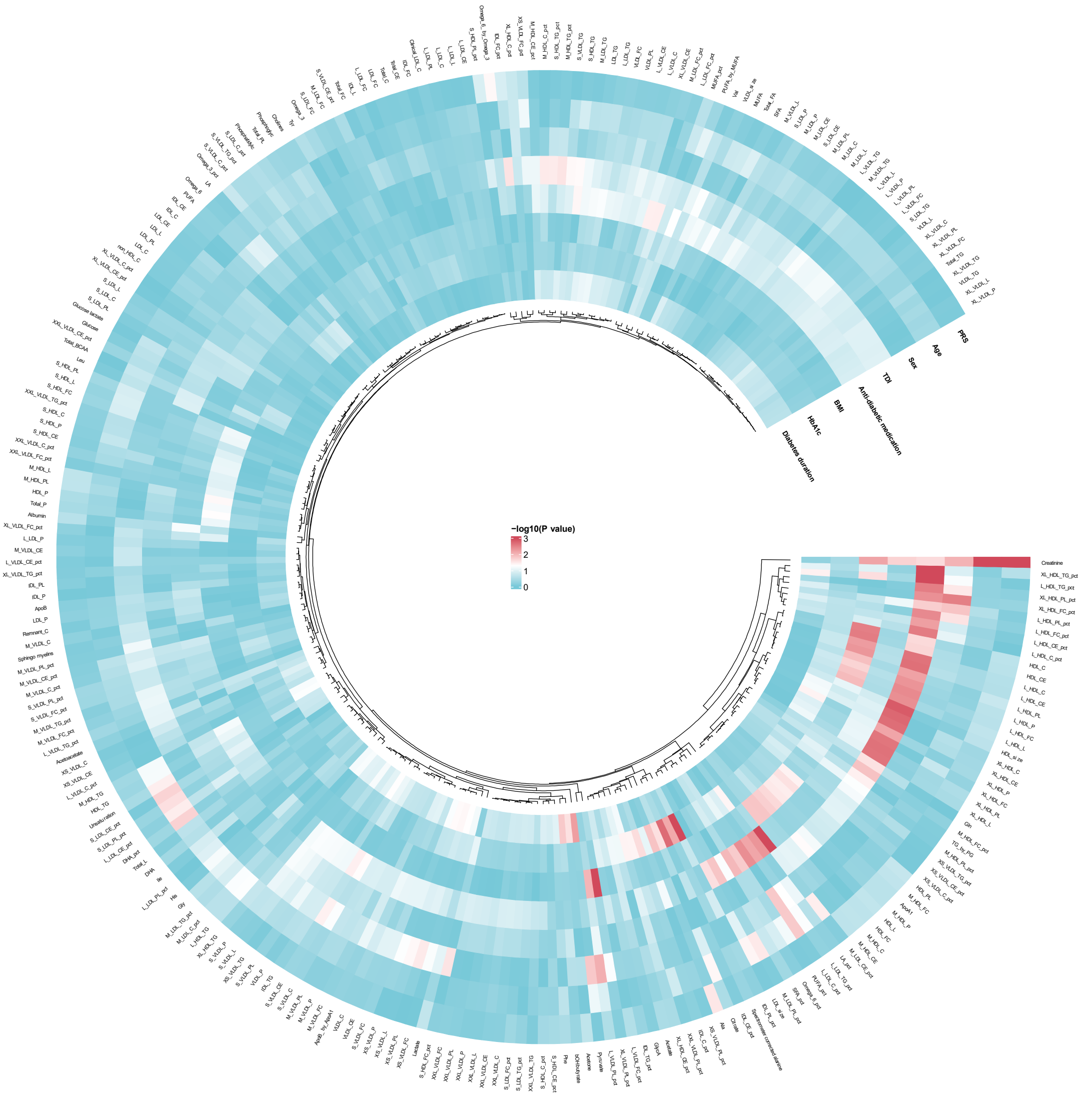

Interaction effects between metabolic biomarkers and genetic and environmental factors based on model 3 was presented in heatmap. The red colour indicates statistical significance, while blue represents non-significance. The intensity of the colour corresponds to the magnitude of the p-value. Clustering patterns are presented using a dendrogram.

Figure S9: Clustering analysis based on PCA dimensionality reduction

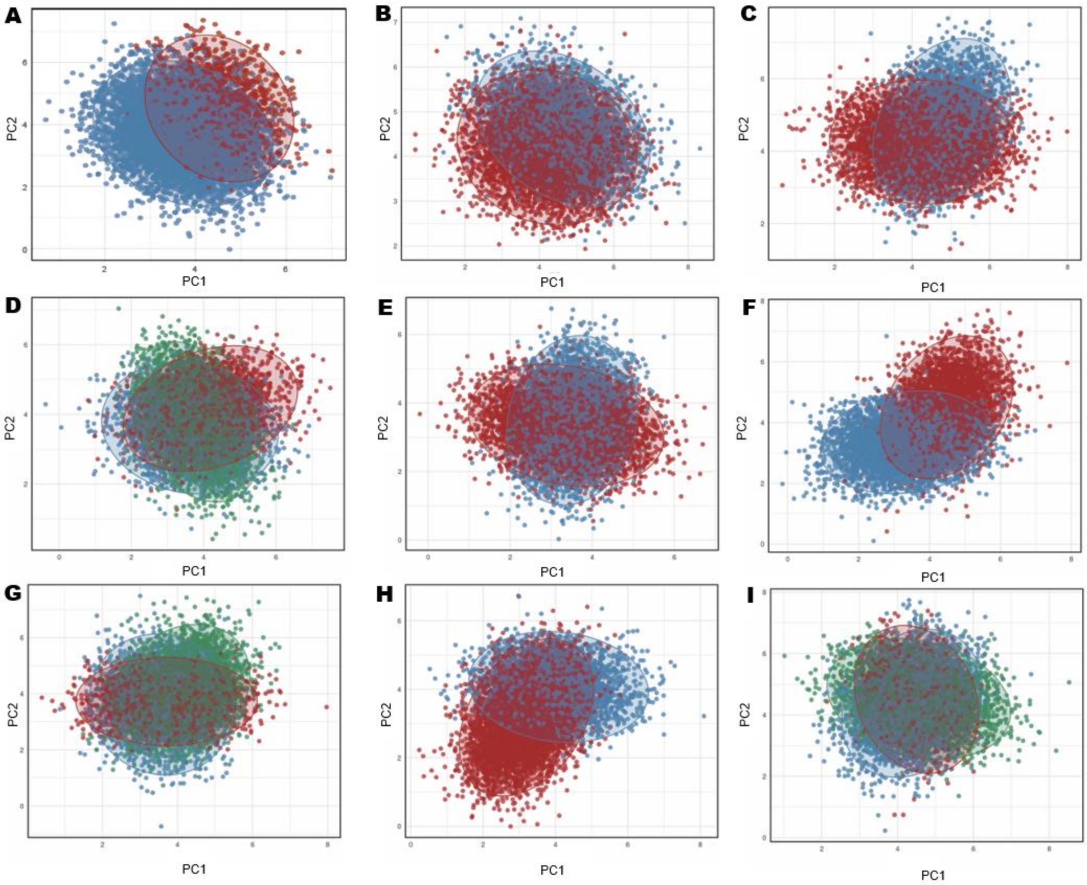

The legends are grouped sequentially as follows: (A) DKD status (Blue: No, Red: Yes), (B) Age (Blue: <60 years, Red: ≥60 years), (C) Gender (Blue: Female, Red: Male), (D) Townsend Deprivation Index (Blue: Q1, Green: Q2–Q3, Red: Q4), (E) BMI (Blue: <30, Red: ≥30), (F) HbA1c (Blue: <53 mmol/mol, Red: ≥53 mmol/mol), (G) TyG Index (Blue: Q1, Green: Q2–Q3, Red: Q4), (H) Anti-diabetic medication use (Blue: No, Red: Yes), (I) Duration of diabetes (Blue: <3 years, Green: 3–10 years, Red: >10 years). PCA, principal component analysis.

Figure S10: Clustering analysis based on OPLS-DA dimensionality reduction

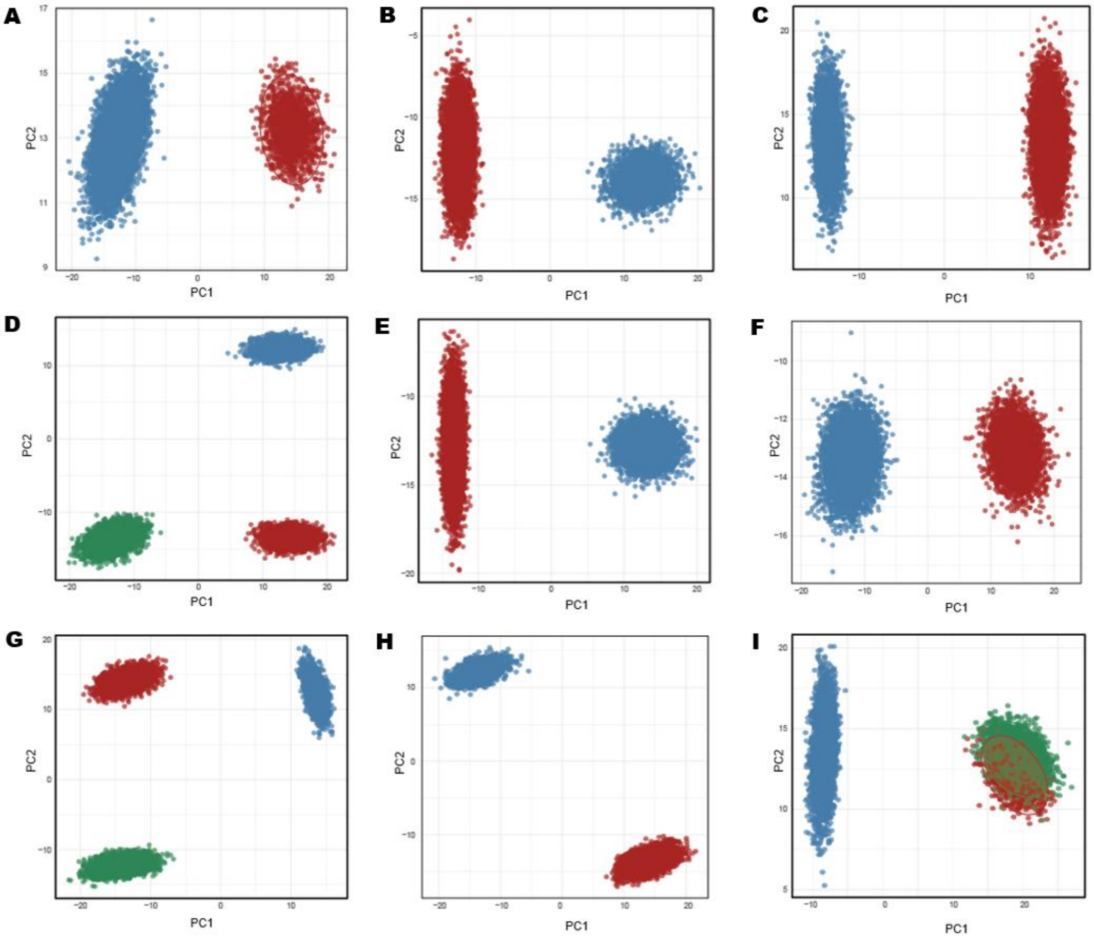

The legends are grouped sequentially as follows: (A) DKD status (Blue: No, Red: Yes), (B) Age (Blue: <60 years, Red: ≥60 years), (C) Gender (Blue: Female, Red: Male), (D) Townsend Deprivation Index (Blue: Q1, Green: Q2–Q3, Red: Q4), (E) BMI (Blue: <30, Red: ≥30), (F) HbA1c (Blue: <53 mmol/mol, Red: ≥53 mmol/mol), (G) TyG Index (Blue: Q1, Green: Q2–Q3, Red: Q4), (H) Anti-diabetic medication use (Blue: No, Red: Yes), (I) Duration of diabetes (Blue: <3 years, Green: 3–10 years, Red: >10 years). OPLS-DA, orthogonal projections to latent structures-discriminant analysis.

Figure S11: Venn diagram of mediating metabolic biomarkers for incidence of DKD progressing to different CKD stages

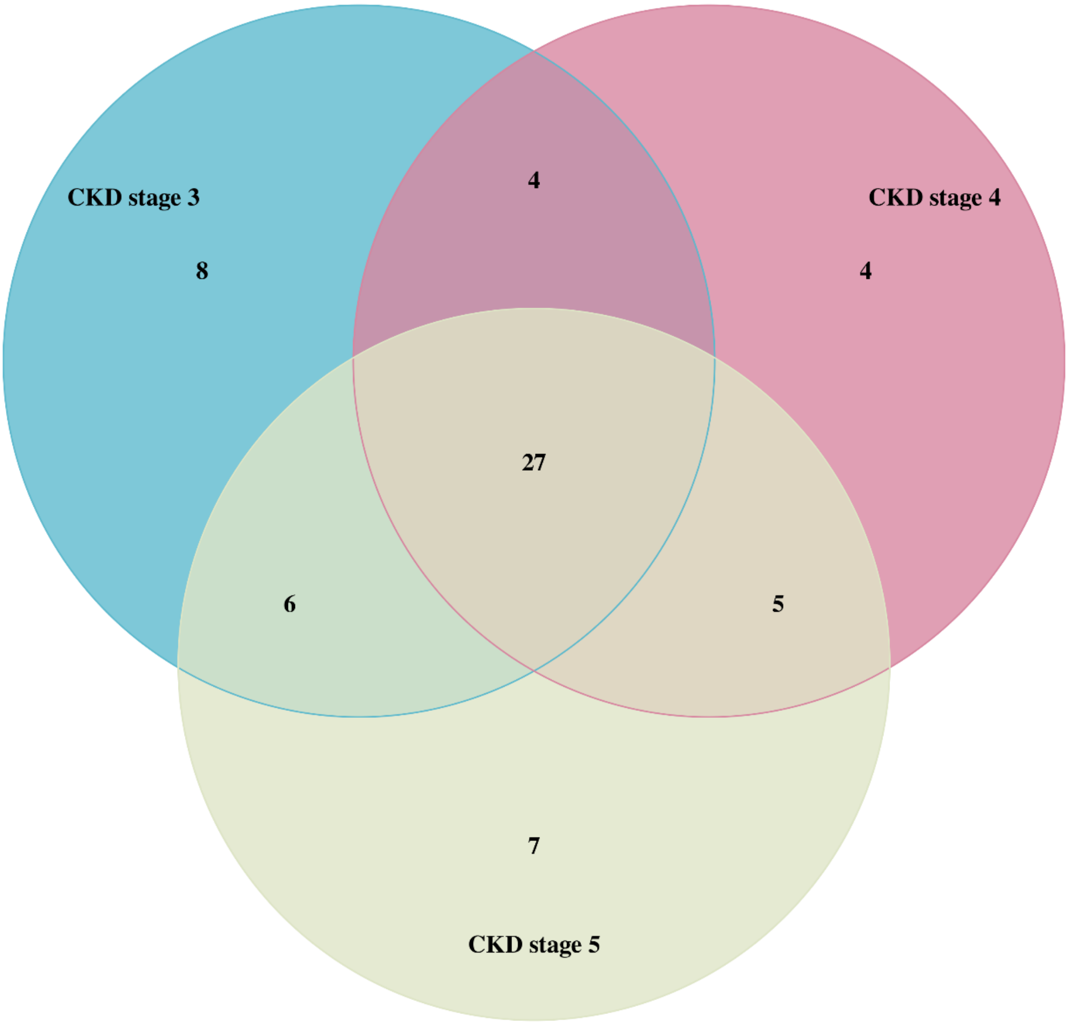

Number of metabolic biomarkers with mediating proportion ≥ 10% were shown. No metabolic biomarkers reached mediating proportion of 10% for DKD progress to CKD stage 1, 2 or end-stage.

Figure S12: Associations between lifestyles and DKD incidence independent of metabolic signatures in observational analysis

Lifestyles	HR (95%CI)	P value
**Model 1**		
Diet score	1.01 (0.97−1.05)	5.9E−1
Sleep duration 7−8 h/d	0.87 (0.80−0.95)	2.4E−3
Physical activity level	0.90 (0.85−0.96)	7.2E−4
Non−current smokers	0.84 (0.72−0.96)	1.3E−2
Moderate alcohol intake	0.87 (0.78−0.96)	7.8E−3
**Model 2**		
Diet score	1.00 (0.97−1.05)	8.2E−1
Sleep duration 7−8 h/d	0.92 (0.84−1.00)	6.4E−2
Physical activity level	0.96 (0.90−1.01)	1.4E−1
Non−current smokers	0.76 (0.66−0.88)	2.4E−4
Moderate alcohol intake	0.90 (0.81−1.00)	4.3E−2
**Model 3**		
Diet score	1.00 (0.96−1.05)	9.2E−1
Sleep duration 7−8 h/d	0.88 (0.80−0.98)	1.7E−2
Physical activity level	0.95 (0.89−1.02)	1.4E−1
Non−current smokers	0.66 (0.56−0.78)	4.0E−7
Moderate alcohol intake	0.88 (0.78−0.99)	3.4E−2

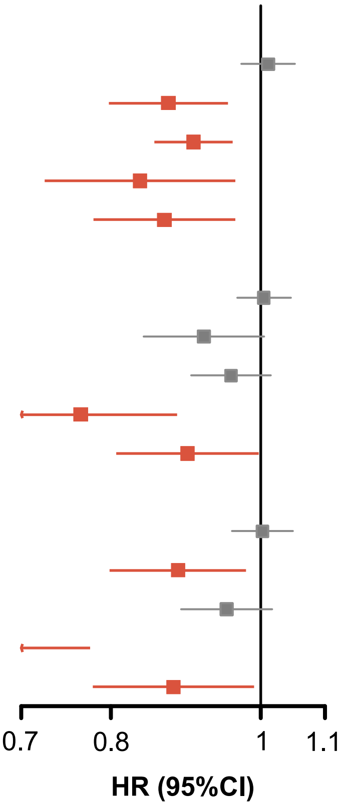

Results were presented as hazard ratio (HR) and 95%CI. Model 1 adjusted for age at recruitment, sex, Townsend deprivation index, ethnicity, and education level (college or university degree). Model 2 additionally adjusted body mass index (BMI), glycated hemoglobin (HbA1c), diabetes duration, hypertension prevalence, use of anti-hyperglycaemic medications, use of antihypertensive medications, and use of lipid-lowering medications. Model 3 included same covariates as model 2, but excluded participants with eGFR < 60.

Figure S13: Associations between lifestyles and DKD mortality independent of metabolic signatures in observational analysis

Lifestyles	HR (95%CI)	P value
**Model 1**		
Diet score	1.02 (0.92−1.14)	6.9×10−1
Sleep duration 7−8 h/d	0.77 (0.61−0.99)	3.9×10−2
Physical activity level	0.84 (0.72−0.98)	2.5×10−2
Non−current smokers	0.65 (0.45−0.92)	1.6×10−2
Moderate alcohol intake	0.75 (0.55−1.01)	6.2×10−2
**Model 2**		
Diet score	1.01 (0.91−1.13)	8.2×10−1
Sleep duration 7−8 h/d	0.85 (0.66−1.08)	1.8×10−1
Physical activity level	0.92 (0.79−1.07)	3×10−1
Non−current smokers	0.58 (0.41−0.82)	2.4×10−3
Moderate alcohol intake	0.79 (0.59−1.08)	1.4×10−1
**Model 3**		
Diet score	1.03 (0.89−1.19)	6.8×10−1
Sleep duration 7−8 h/d	0.84 (0.61−1.15)	2.8×10−1
Physical activity level	0.89 (0.73−1.09)	2.5×10−1
Non−current smokers	0.37 (0.24−0.56)	4.8×10−6
Moderate alcohol intake	0.66 (0.43−1.00)	5.1×10−2

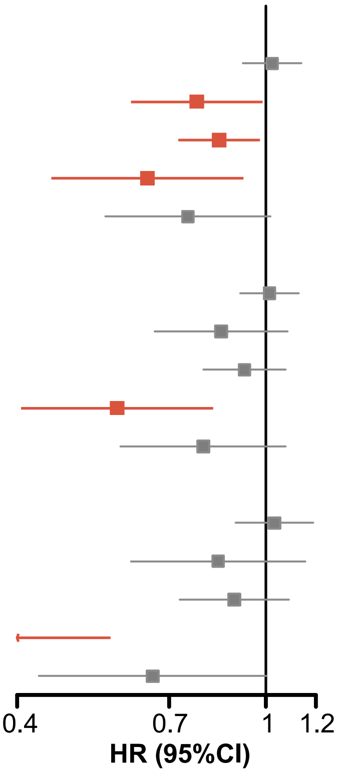

Results were presented as hazard ratio (HR) and 95%CI. Model 1 adjusted for age at recruitment, sex, Townsend deprivation index, ethnicity, and education level (college or university degree). Model 2 additionally adjusted body mass index (BMI), glycated haemoglobin (HbA1c), diabetes duration, hypertension prevalence, use of anti-hyperglycaemic medications, use of antihypertensive medications, and use of lipid-lowering medications. Model 3 included same covariates as model 2, but excluded participants with eGFR < 60.

Figure S14: Venn diagram of enriched pathways for lifestyle-associated metabolic biomarkers

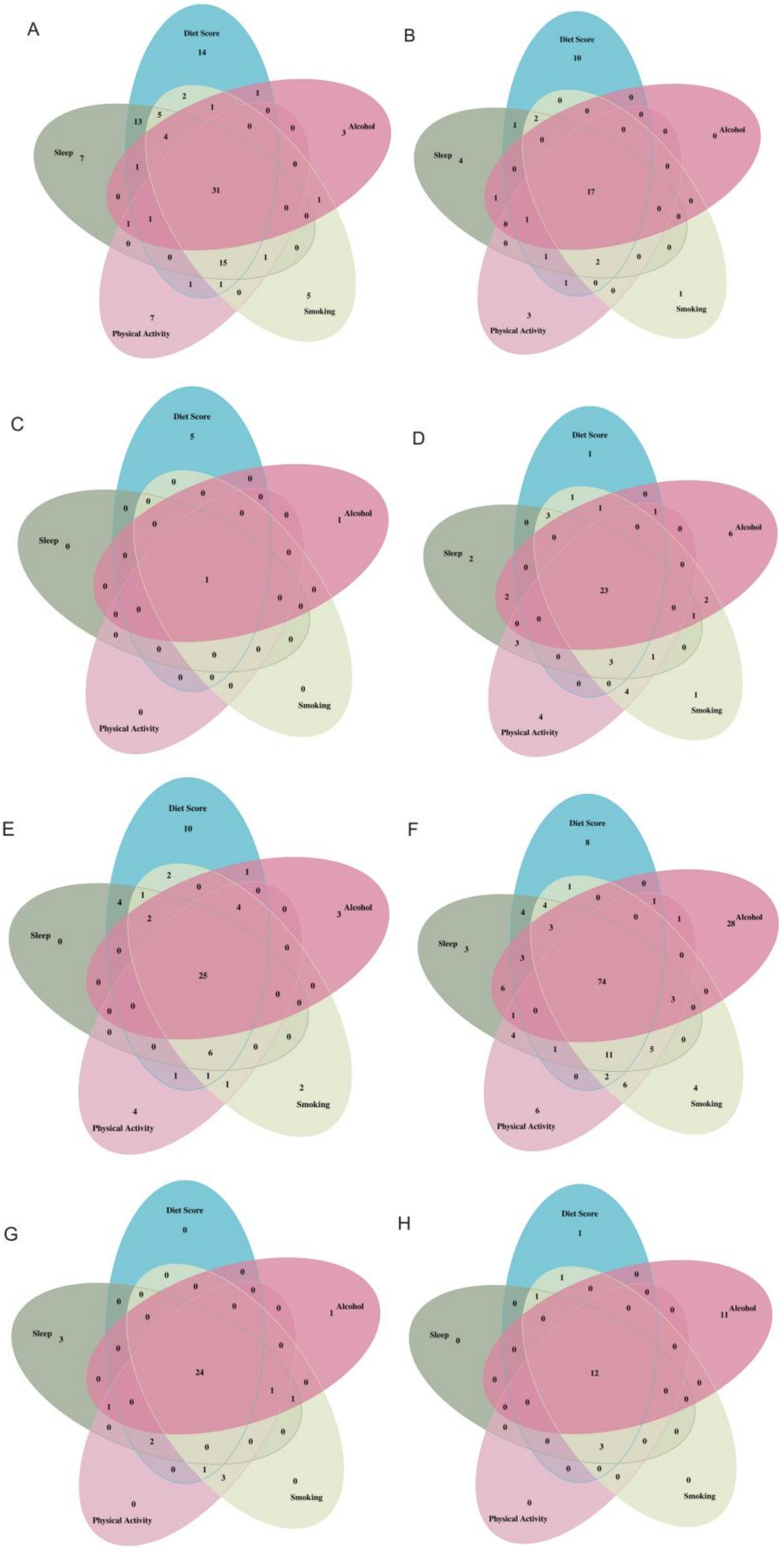

A. BioPlanet 2019; B. KEGG 2021 Human; C. MsigDB Hallmark 2020; D. Reactome 2022; E. WikiPathways 2024 Human; F. GO Biological Process 2023; G. GO Cellular Components; H. GO Molecular Function.

Figure S15: Gene enrichment analysis based on BioPlanet 2019 database

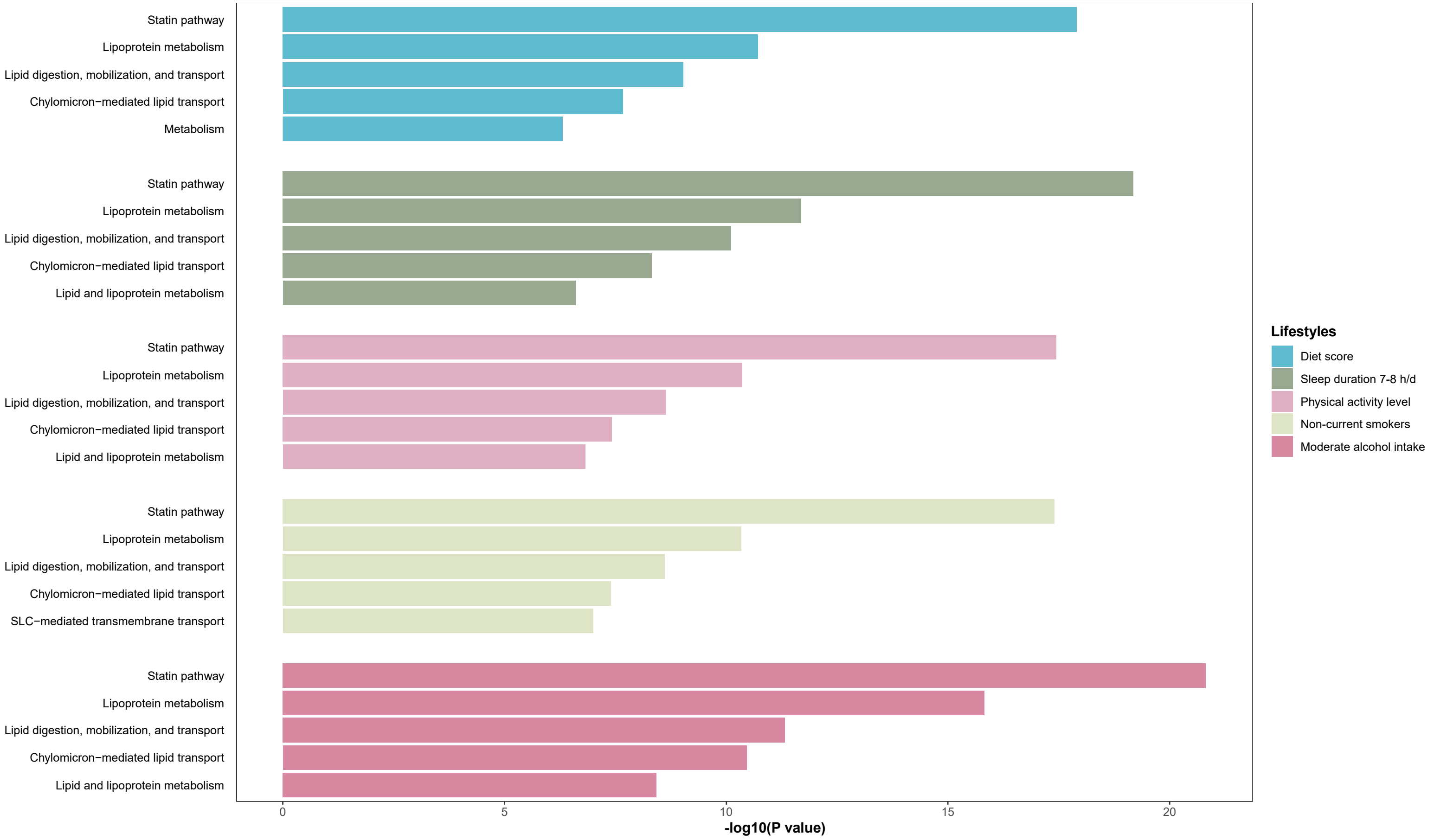

only the top five results with P-values from small to large were displayed

Figure S16: Gene enrichment analysis based on MsigDB Hallmark 2020 database

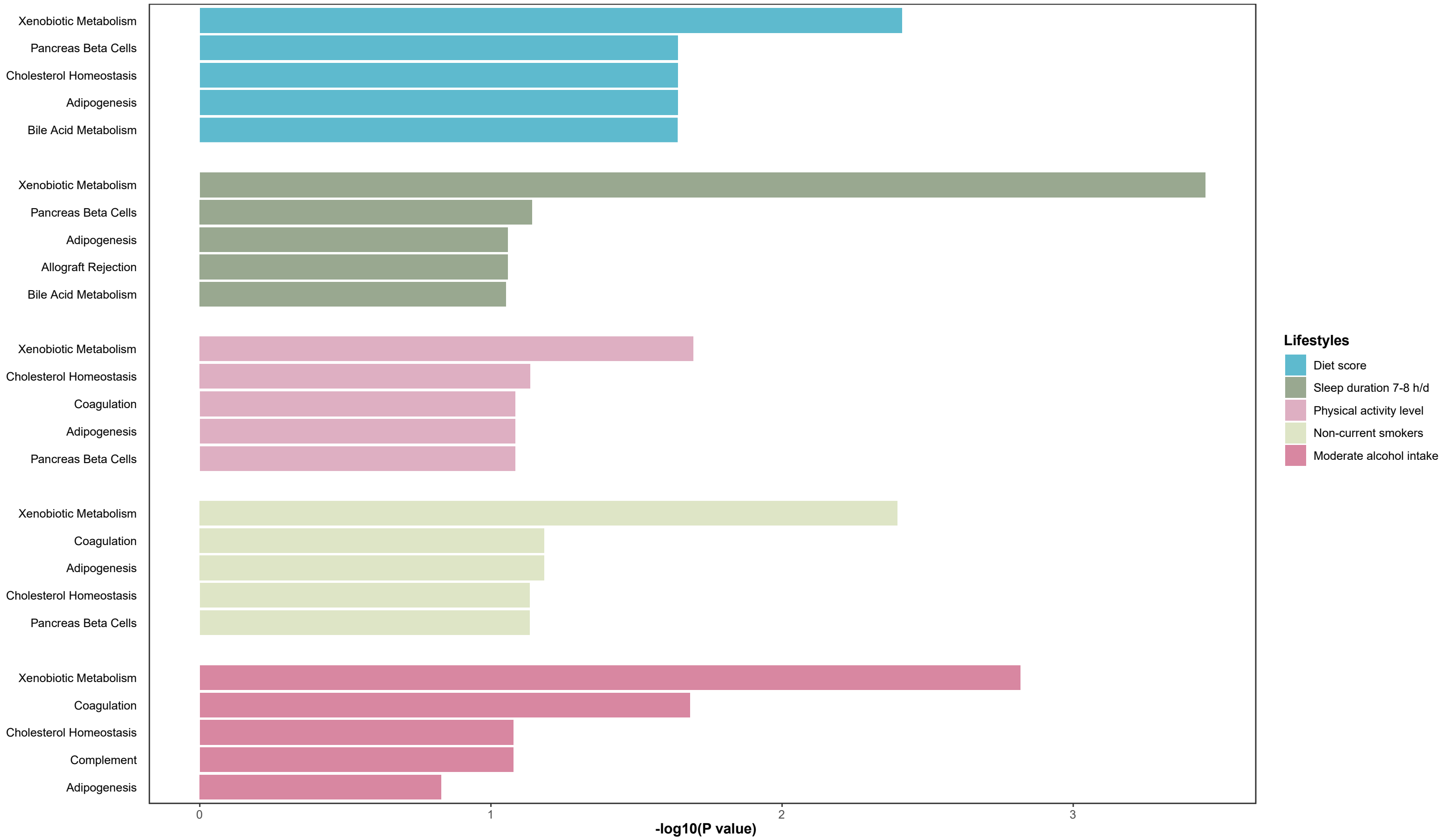

only the top five results with P-values from small to large were displayed

Figure S17: Gene enrichment analysis based on Reactome 2022 database

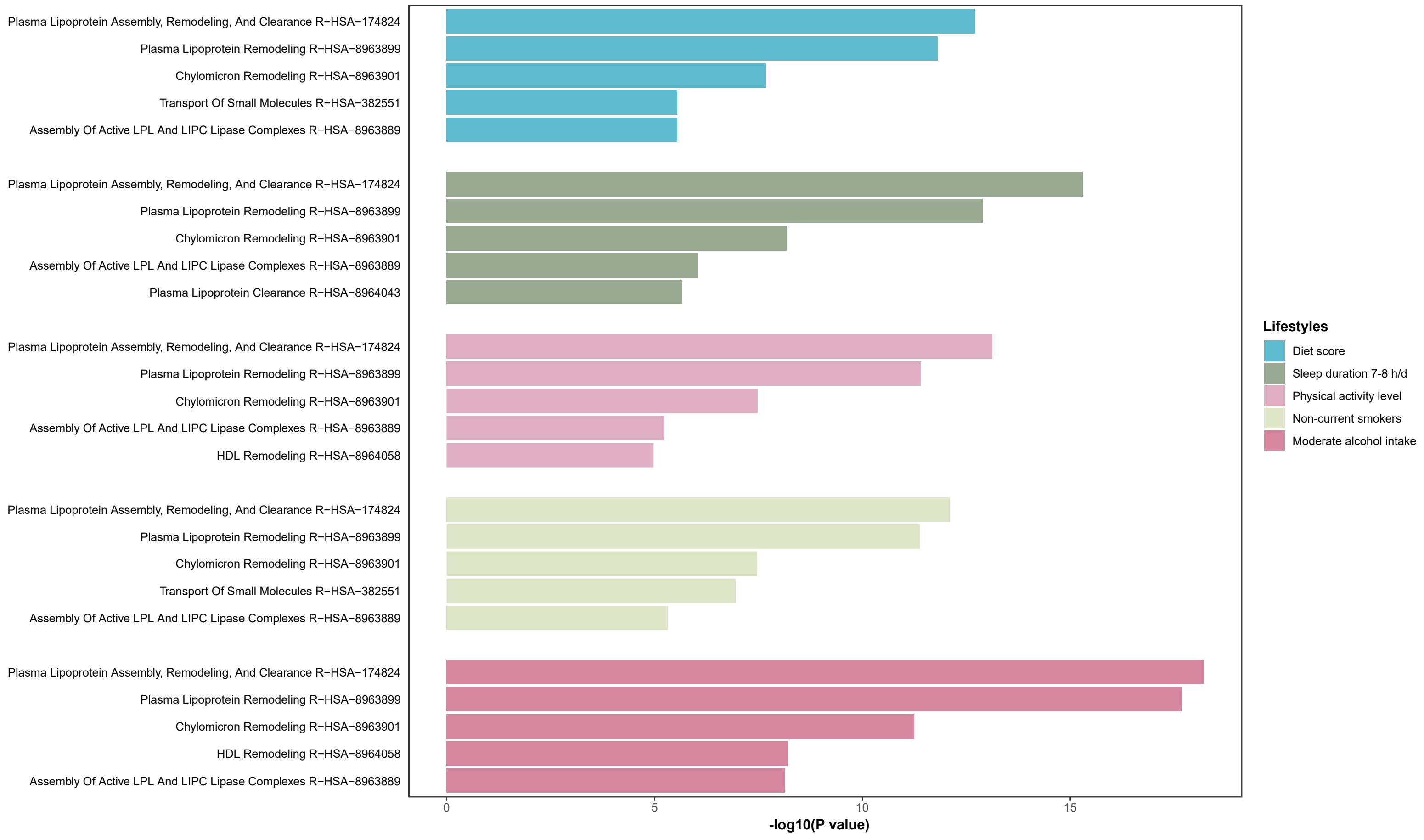

only the top five results with P-values from small to large were displayed

Figure S18: Gene enrichment analysis based on WikiPathways 2024 Human database

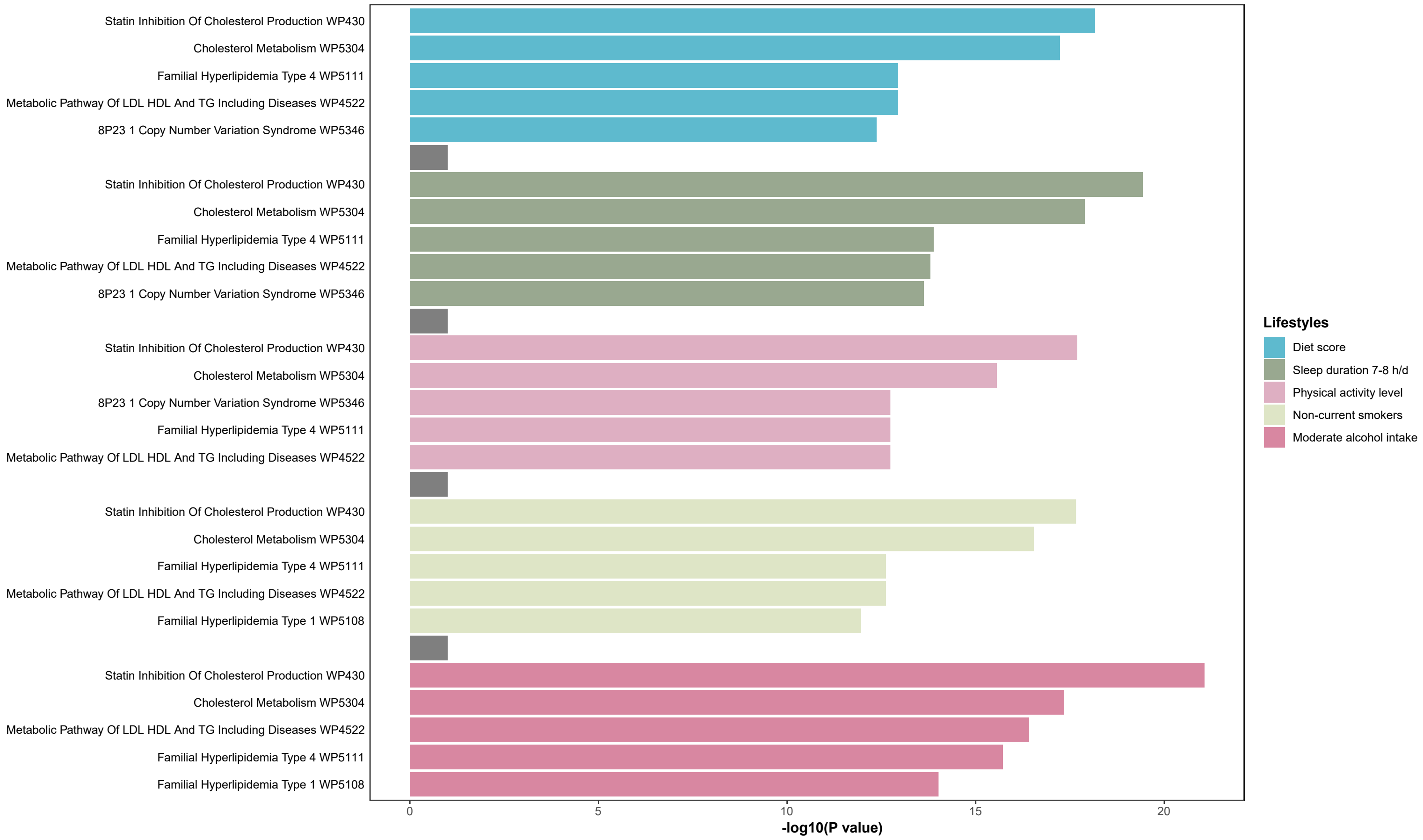

only the top five results with P-values from small to large were displayed

Figure S19: Gene enrichment analysis based on GO Biological Process 2023 database

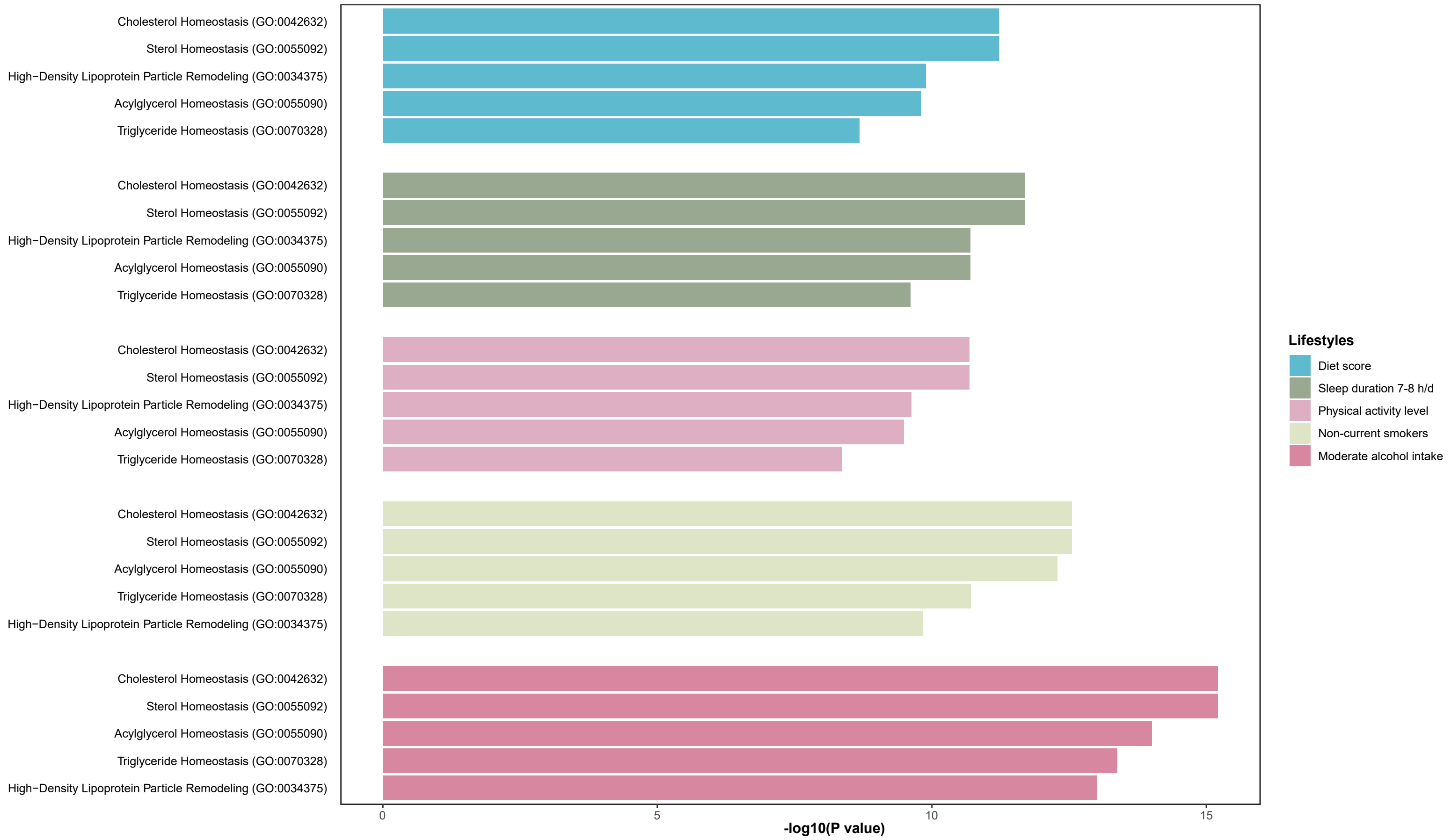

only the top five results with P-values from small to large were displayed

Figure S20: Gene enrichment analysis based on GO Cellular Component 2023 database

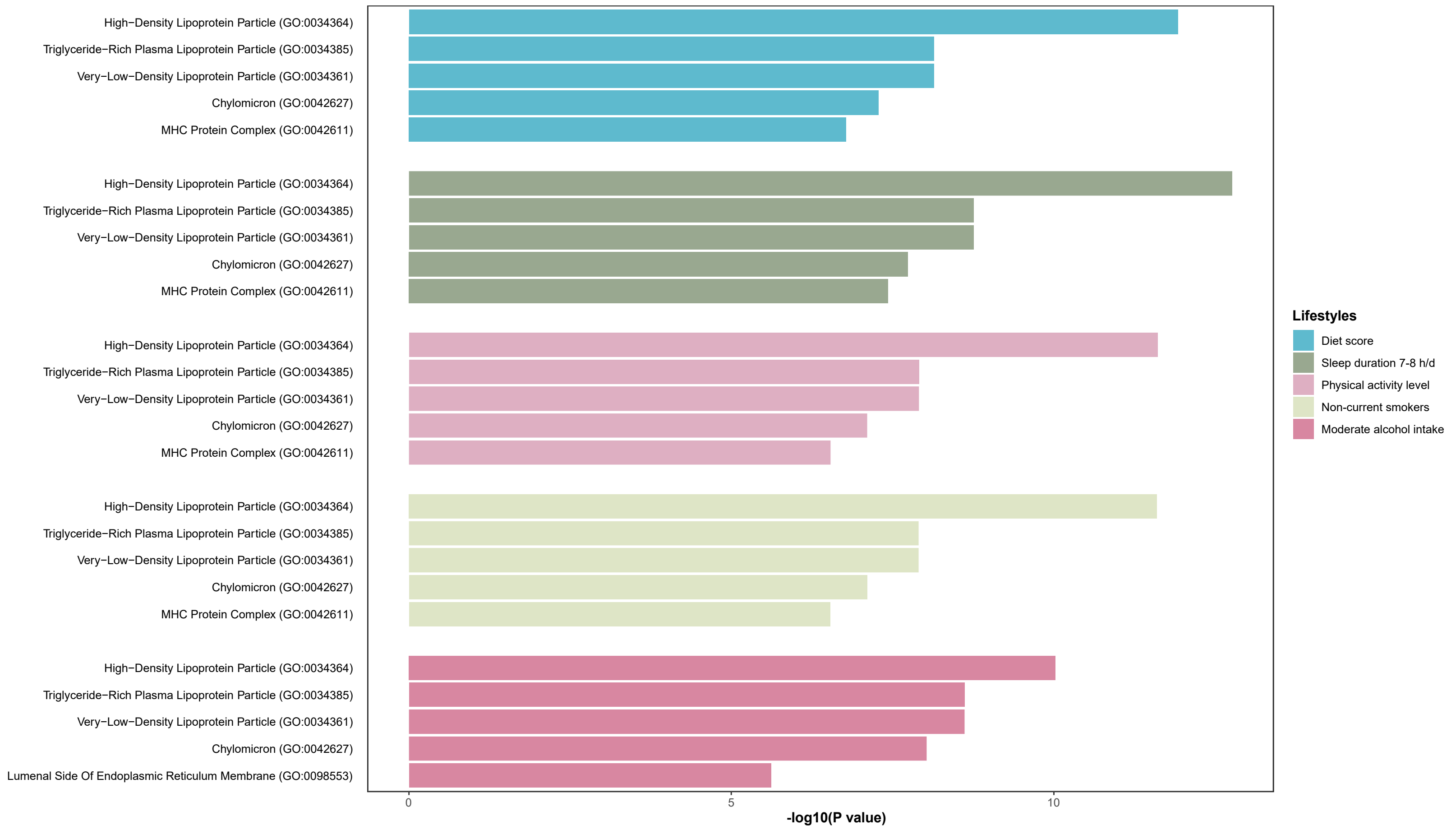

only the top five results with P-values from small to large were displayed

Figure S21: Gene enrichment analysis based on GO Molecular Function 2023 database

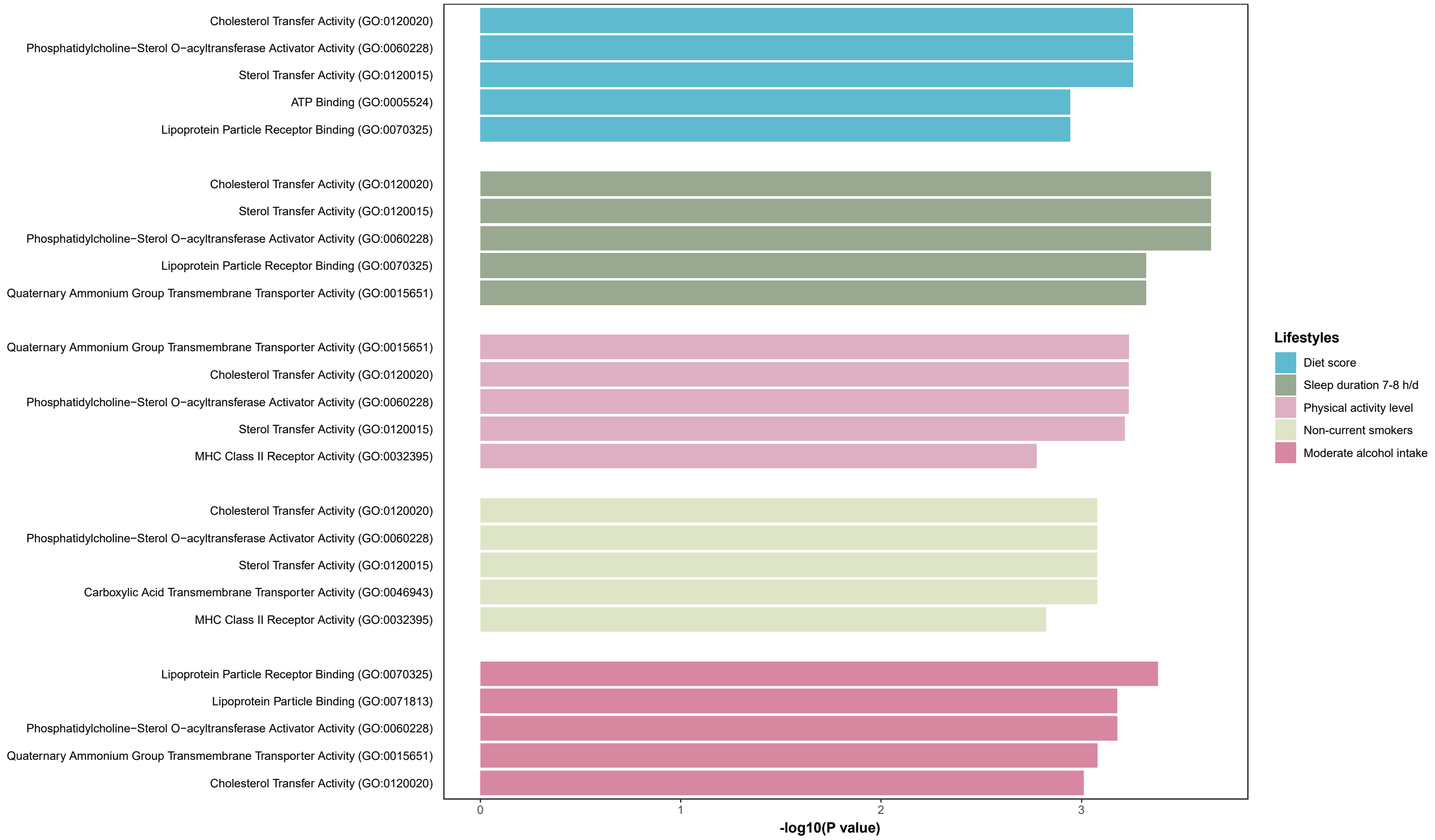

only the top five results with P-values from small to large were displayed

Figure S22: Gene enrichment analysis based on GTEx Tissues V8 2023 database

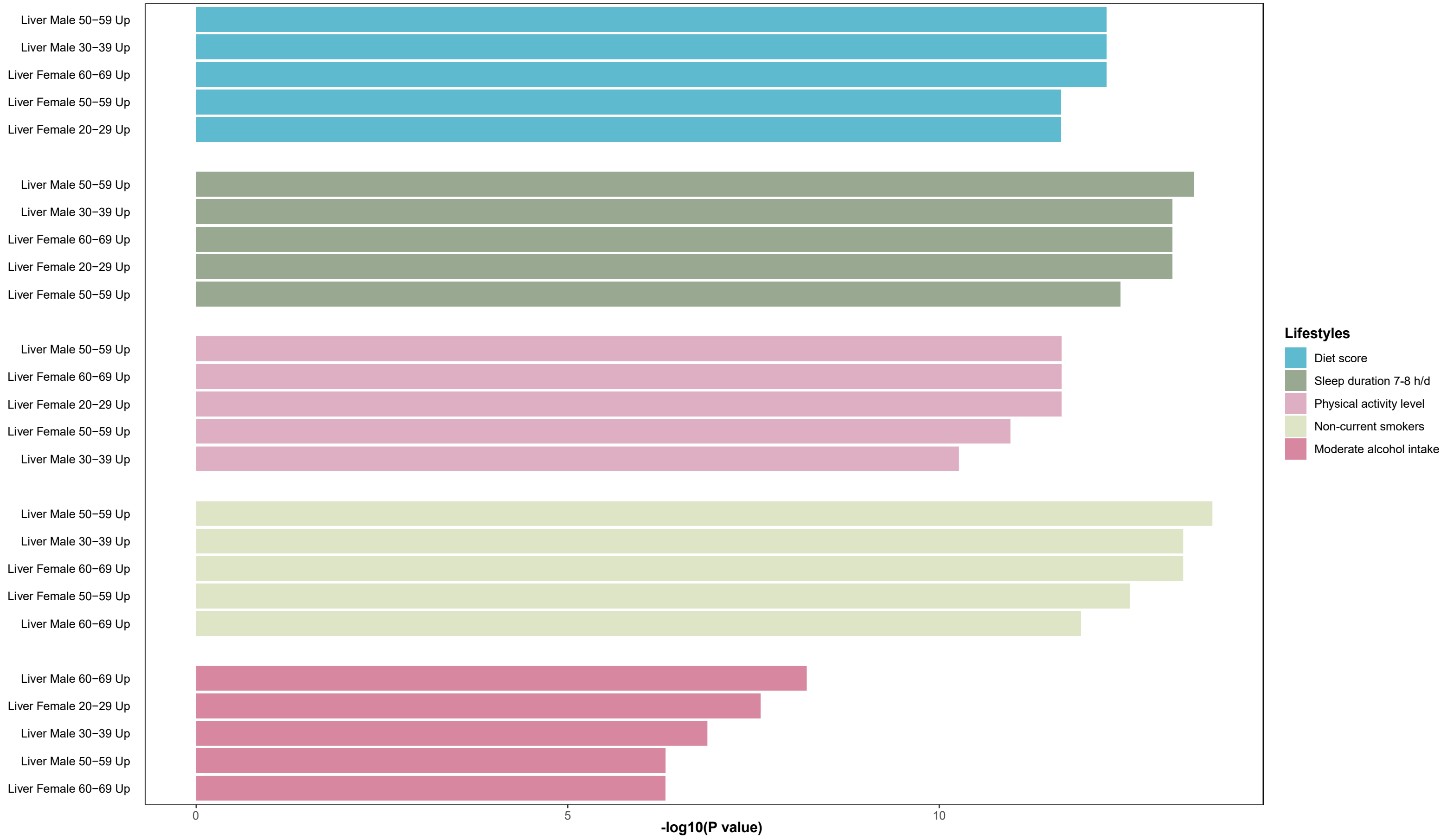

only the top five results with P-values from small to large were displayed

Figure S23: Mendelian randomization analysis with metabolic biomarkers as exposures and DKD or eGFR as outcomes

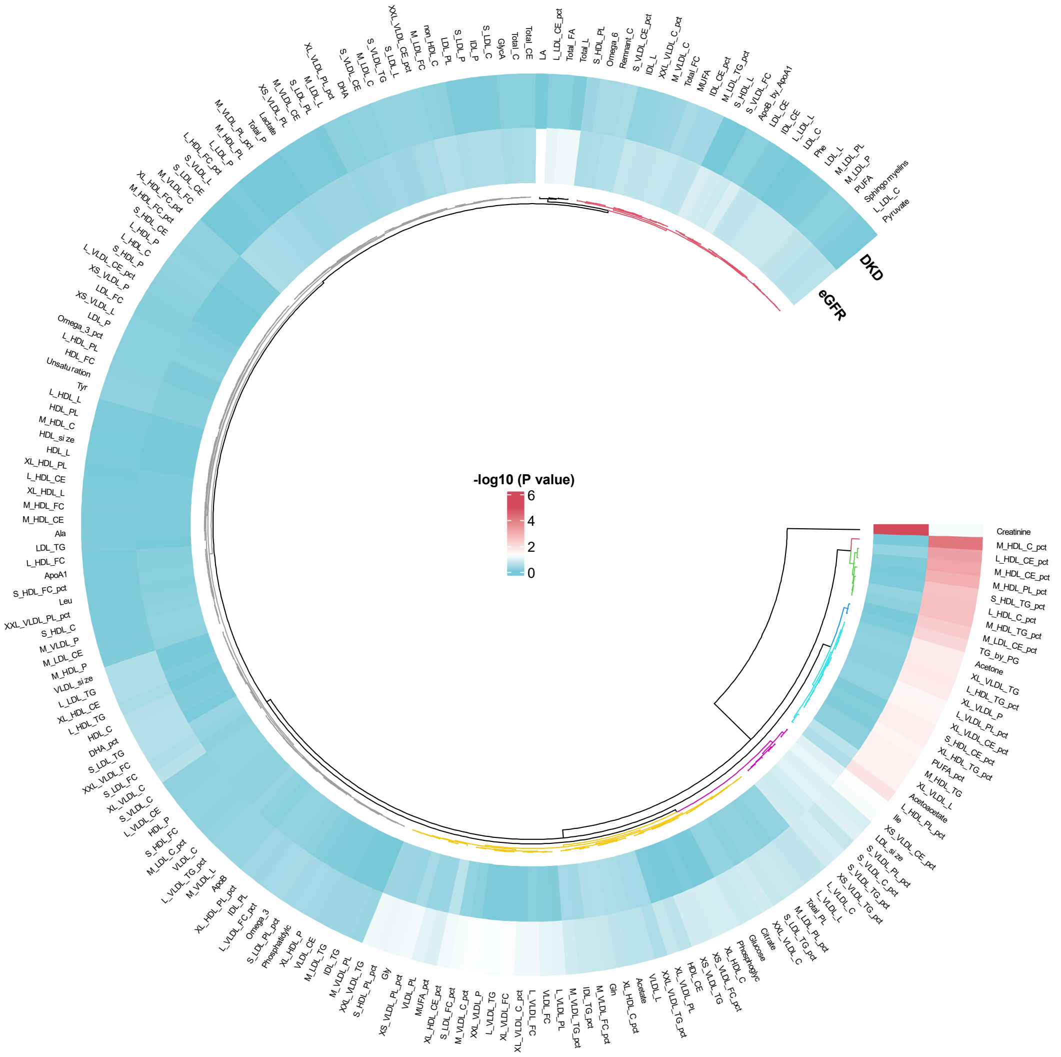

The red colour indicates statistical significance, while blue represents non-significance. The intensity of the colour corresponds to the magnitude of the p-value. Clustering patterns are presented using a dendrogram.

Figure S24: Mendelian randomization analysis with DKD or eGFR as exposures and metabolic biomarkers as outcomes

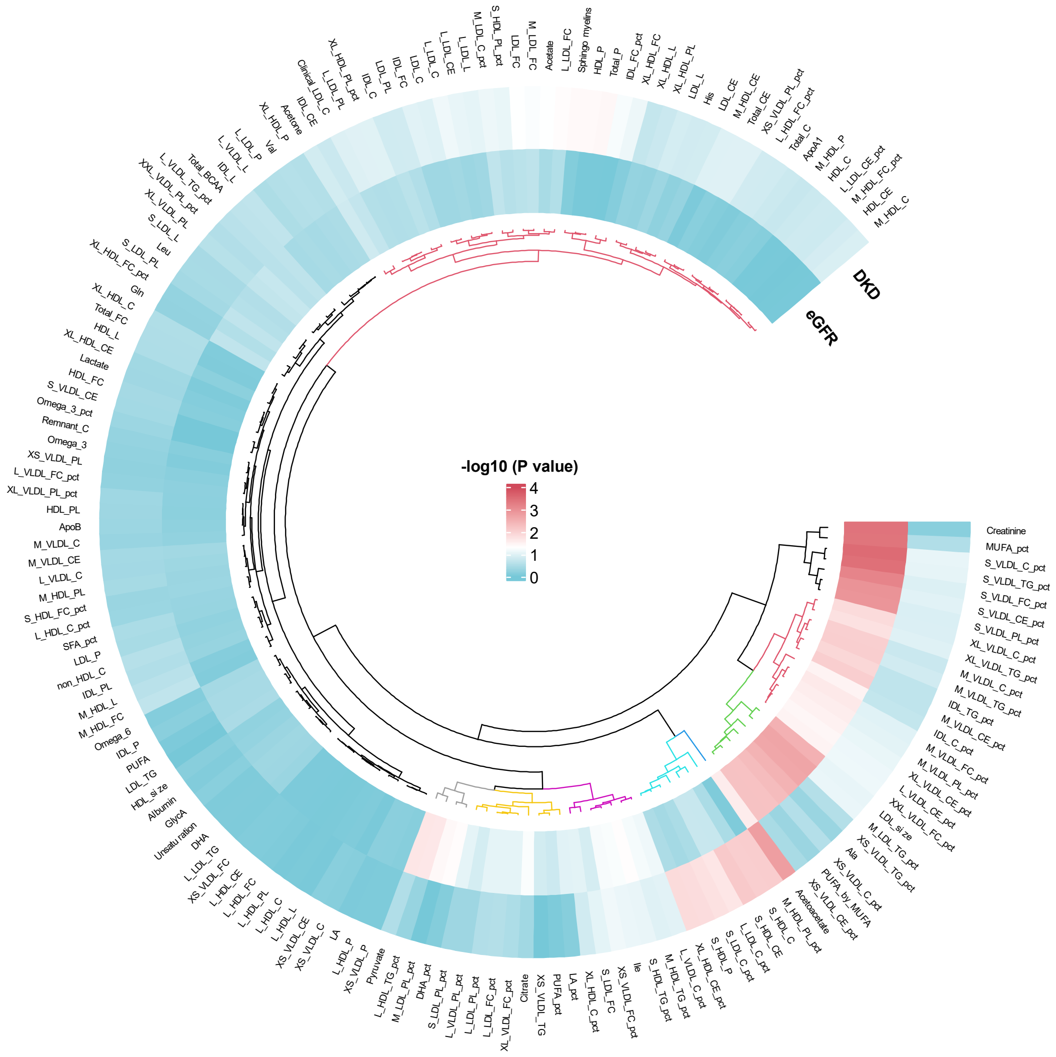

The red colour indicates statistical significance, while blue represents non-significance. The intensity of the colour corresponds to the magnitude of the p-value. Clustering patterns are presented using a dendrogram.
